# Mental illness, crime and mental health resources: insights from Taiwan using text analysis and spatio-temporal analysis

**DOI:** 10.1136/bmjph-2025-002832

**Published:** 2026-05-06

**Authors:** Chia-Jung Lin, Meng-Jung Lin, Jihn-Fa Jan

**Affiliations:** 1 Department of Land Economics, National Chengchi University, Taipei City, Taipei City, Taiwan; 2 Department of Sociology, National Taiwan University, Taipei City, Taiwan

**Keywords:** Public Health, Mental Health, Data Collection

## Abstract

**Introduction:**

In the aftermath of notable criminal incidents, discussions surrounding mental illness and public safety have gained prominence. This study investigates the association between mental illness and crime in Taiwan and explores how mental health resources are associated with crime patterns across space and time.

**Methods:**

We analysed criminal legal judgements in Taiwan using natural language processing to identify cases involving indications of mental illness based on court records. Geographic Information System techniques and Geographical and Temporal Weighted Regression (GTWR) were used to examine spatiotemporal patterns and associations between mental health resources and crime.

**Results:**

By examining legal judgements, we estimate that about 0.4% of crimes involve individuals with indications of mental illness, based on what is recorded in court decisions. Spatiotemporal analysis and GTWR showed that areas with higher mental health resources often aligned with higher crime rates for certain offences, such as violent crimes and larceny, reflecting urbanisation and victim support needs. Conversely, a greater density of psychiatry departments and doctors was associated with reductions in crimes involving mental illness.

**Conclusions:**

Crimes involving individuals with mental illness account for a small fraction of all criminal cases in Taiwan. These findings highlight the potential role of mental health resources in mitigating certain types of crime and contribute to understanding the complex relationship between mental health and public safety.

**WHAT IS ALREADY KNOWN ON THIS TOPIC**

**WHAT THIS STUDY ADDS**

- This study estimates that crimes involving individuals with indications of mental illness account for about 0.4% of total crimes in Taiwan. Using natural language processing (NLP) and Geographical and Temporal Weighted Regression (GTWR), it reveals that higher psychiatry resource density is associated with reductions in specific crimes, while urbanisation and victim support needs may explain some positive correlations.

**HOW THIS STUDY MIGHT AFFECT RESEARCH, PRACTICE OR POLICY**

- The findings suggest that strategically increasing targeted mental health resources, such as psychiatry departments and doctors, could reduce certain crimes and improve public safety.

## Introduction

 Discussions around mental illness have increasingly intersected with public safety concerns, particularly in the aftermath of high-profile crimes. Research has long shown that public perceptions tend to overestimate the likelihood of violence among individuals with mental illness,^1^,^3^ even though only a modest proportion of violence is attributable to mental disorders. Meta-analyses and large-scale studies consistently report that psychosis explains only a limited share of violent incidents in the population.^4 5^ These findings contrast sharply with public narratives that often emerge after violent events.

Across countries, public concern about mental illness and crime is often shaped by a small number of highly visible cases, despite their rarity. Similar patterns are observed in Taiwan. At the same time, the prevalence of mental disorders in Taiwan has increased substantially in recent decades,^6^ highlighting the importance of examining the broader social implications of mental illness. Taiwan’s extensive administrative and legal data therefore offer a valuable opportunity to study the relationship between mental illness, crime and mental health resources.

Despite the public discourse, research challenges the common perceptions that mental illness is a predominant driver of crime. While specific mental disorders, such as substance abuse and violence, are associated with increased criminal behaviours, these disorders account for only a small percentage of overall violence.^7 8^ For example, only 1–5% of violence can be attributed solely to mental illness.^7^ Even among offenders with schizophrenia-spectrum disorders or substance dependence, violence makes up only a minority of criminal acts.^8 9^ One study found that only 7.5% of crimes among offenders with serious mental illness were directly related to their mental health symptoms.^10^ Similarly, mentally abnormal homicides represent just 3.7% of murder cases, while the impact from mental healthcare utilisation or incarceration rates was minimal.^11^

Mental health disparities are shaped by neighbourhood conditions, with factors like socioeconomic disadvantage, crime, noise and congestion linked to poor mental health.^12 13^ Similarly, crime patterns often reflect concentrated disadvantage. Poverty, social isolation and weakened social controls foster environments conducive to crime.^14^ Institutional deficiencies, norms like the ‘code of the street’, and socioeconomic conditions of surrounding areas perpetuate cycles of violence.^15 16^ These findings collectively emphasise how structural and geographic inequalities shape both mental illness and crime.

To mitigate risks associated with mental illness, research points to the importance of treatment and mental healthcare. Comorbid substance abuse amplifies risks of violence among individuals with mental illnesses,^17 18^ while interventions like access to treatment facilities significantly reduce violent crimes such as homicides and assaults by addressing underlying drug dependency and systemic violence.^19^ Meanwhile, access to mental healthcare also helps reduce crime. Psychotropic drug use has been linked to lower violent crime rates,^20^ and greater availability of mental healthcare offices has been associated with reduced overall crime while addressing social costs.^21^ Furthermore, expanding Medicaid for mental health services has been shown to lower incarceration rates among low-income populations, demonstrating the broader benefits of integrating mental healthcare into public safety strategies.^22^

Due to the lack of readily available information, the accurate number of crimes attributable to mental illnesses in Taiwan is unclear. To address this gap, this study uses natural language processing (NLP) techniques to analyse court documents, identifying mentions of Criminal Code Article 19—which allows for reduced sentences for individuals with mental disorders—as a proxy indicator for crimes associated with mental illness. While this method is newly developed and offers only an approximate estimate, it provides an initial quantification of the proportion of crimes potentially linked to mental health issues. NLP has been increasingly used in legal document analysis to extract meaningful information from unstructured text. For example, recent studies in the legal domain have demonstrated the potential of these tools for processing complex judicial texts and identifying relevant patterns.^23^,^25^ By using NLP in our study, we aim to provide a preliminary understanding of the relationship between mental illness and crime in Taiwan.

Motivated by ongoing discussions about mental illness, crimes and advancements in NLP, coupled with access to rich administrative data, this study seeks to explore the relationship between mental illness and crime. The primary aim is twofold: first, to examine the association between mental illness and criminal cases, and second, to explore the relationship between the availability of mental health resources and crime hot spots. We hypothesise that regions with better access to mental health resources will have lower crime rates. This hypothesis forms the basis for analysing how mental health resource accessibility influences crime prevalence across administrative regions in Taiwan.

The study addresses the following research questions:

1. What proportion of criminal cases can be attributed to individuals with mental illness?
2. What are the trends in the spatio-temporal distribution of criminal cases involving individuals with mental illness?
3. What is the association between the availability of mental health resources and crime hot spots? Are there additional social factors that exhibit a connection?

By addressing societal stigmatisation and discrimination, the research aims to inform discussions about the social and legal contexts surrounding mental illness and crime.

## Data and methods

### Data

#### Data on mental health and crime

This study uses data from various official sources, including legal judgements, crime statistics, medical resource information and regional socioeconomic characteristics. To estimate the number of crimes attributed to people with mental illness, legal judgement data were obtained from the Judicial Yuan Open Data Platform.^26^ The complete dataset, covering judgements from summary courts to the Supreme Court, has been available since 2001. This study uses the latest update as of 22 November 2023, analysing judgements from Taiwan for the period 2001 to 2021.

To examine the spatial distribution of crimes related to mental illness, we acquired organised data from the National Police Agency within the Ministry of the Interior, which includes the address, telephone number, longitude and latitude information for each police station.^27^ Additionally, to facilitate comparisons between crimes committed by individuals with mental illness and those without, this study used annual statistics on selected crime types reported in each county and city.^28^ These data were sourced from the Statistics of Criminal Investigation and Crime Prevention provided by the Ministry of the Interior Statistics Department.

#### Data on mental health resources

To measure mental health resources, we used the Statistics on the Current Status of Medical Institutions.^29^ These data, provided annually since 2012 by the Statistics Division of the Ministry of Health and Welfare, document the distribution of medical resources across administrative regions. The dataset includes five key categories: hospital beds statistics, hospital workforce statistics, hospital department statistics, clinic workforce statistics and clinic department statistics. It also includes information on beds designated for acute and chronic mental illnesses, as well as psychiatric intensive care beds available in each region.

The workforce statistics detail the number of clinical psychologists, counselling psychologists and clinical social workers in each administrative area, while department statistics specify the number of psychiatry services offered in hospitals and clinics. In cases where the Ministry of Health and Welfare lacks information on the number of practising physicians by discipline within administrative regions, supplemental data were obtained from the National Federation of Medical Doctor Associations of Taiwan.^30^ Since 1998, this federation has consistently compiled statistics on practising physicians and medical institutions in Taiwan.

By integrating these data with regional population and calculating the population density of resources, we developed indices summarising the availability of mental health resources in each region.

#### Data on socioeconomic characteristics of administrative regions

As previous research suggests,^12 13 31^ the socioeconomic status of an area is an important risk factor for both mental illness and crime. To account for this, we incorporated data on the number of low-income households in each administrative district and the proportion of individuals aged 15 and above with a college degree.^32 33^ These data were sourced from the SocioEconomic Database of Geographic Information System, compiled by the Ministry of the Interior.

### Methods

#### Text analysis

##### Create mental illness crime database

Judgements from the Judicial Yuan Open Data Platform were stored in JSON format, containing information such as JID (Judgment ID), JYEAR (Case Year), JCASE (Judgment Type), JNO (Judgment Number), JDATE (Judgment Date), JTITLE (Crime Type) and JFULL (Full Description of the Judgment). Given the large volume of judgements spanning over 11 years, we identified those referencing Criminal Code Article 19 as related to mental illness. Additionally, since the same case could undergo multiple judgements, we extracted judgements with phrases like ‘Disobey (不服) {JID} …。’ or judgements indicating recurrence in the courts. Using regular expressions, past JIDs were extracted and linked to the judgements, enabling a more precise analysis.

The rationale for using Criminal Code Article 19 is that it stipulates an offence is not punishable (Paragraph 1) or is subject to a reduced sentence (Paragraph 2) if the offender, due to a mental disorder or defect, lacked the capacity to judge their actions or had a significantly diminished capacity at the time of the offence. In judicial practice, this determination relies on court-mandated forensic psychiatric evaluations. Such evaluations typically involve severe mental disorders, including schizophrenia-spectrum disorders, bipolar disorder, delusional disorders, intellectual disabilities and certain neurocognitive impairments.^34^

Article 19 operates as a legal criterion rather than a measure of the overall prevalence of mental illness among offenders. It reflects only cases in which mental illness was raised during legal proceedings and formally evaluated, and therefore likely underrepresents individuals with more common conditions such as depression or anxiety. Although validating these judicial assessments with clinical data would provide a more complete picture, linking identifiable court judgements with the National Health Insurance Research Database (NHIRD) is not feasible under current data-governance protocols. NHIRD data are de-identified and encrypted, preventing direct linkage to individuals named in court documents without special government authorisation that is not available for this study.^35^ For these reasons, our analysis relies on information recorded in legal judgements as the most accessible proxy for identifying cases involving mental illness.

##### Obtain geographic information of judgements

Geographic information was extracted from the reported police office. Patterns such as ‘the sue is reported from (訴由) {Police Office} …。’ or ‘the case is reported from (案經) {Police Office} …。’ were used to identify the police office initially. Short names of police offices were matched to their possible full names, as identical names may refer to different locations. County and city names mentioned in the judgements were then identified to determine the correct police office. Finally, manual checks were conducted to resolve duplicate police office names.

##### Text preprocessing

Because Chinese legal texts are written without whitespace, word segmentation is necessary for accurate text analysis. Simple keyword searches can produce substantial misclassification due to segmentation ambiguity. To address this, we used CkipTagger, an open-source Chinese NLP tool developed by Academia Sinica,^36^ to perform word segmentation, part-of-speech tagging and named entity recognition. This approach allows clinically and legally relevant terms to be distinguished from incidental narrative expressions in judgement texts. Detailed descriptions of the NLP pipeline and tool performance are provided in online supplemental appendix S1.1–S.1.3.

### Spatiotemporal analysis

After extracting the locations of mental-illness-related crimes, we used ArcGIS to analyse and visualise the spatiotemporal features of the data. The spatiotemporal analysis involved creating a space-time cube from defined locations to summarise observations per time period or calculate summary statistics for geographical locations across time. The cube contained annual counts for each district from 2012 to 2021, resulting in 10 time steps and 365 locations, for a total of 3650 space-time bins.

We then conducted an emerging hot spot analysis to detect spatial and temporal trends. For each bin, the analysis computes the Getis–Ord Gi* statistic and corresponding z-score and false discovery rate (FDR)-adjusted p value, using a contiguity-based spatial weights matrix that includes both edge and corner neighbours. Bins with statistically significant Gi* values at p<0.05 after FDR correction are classified as hot spots or cold spots. For each location, the tool applies the Mann-Kendall trend test to the sequence of Gi* z-scores across all time steps to assess whether the intensity of clustering increases, decreases or remains stable over time.

Locations that do not meet the significance criteria for any of these categories are left unclassified by the tool. In the maps presented in this study, these locations appear as not applicable (NA), indicating the absence of statistically significant spatiotemporal clustering rather than missing data. Emerging hot spot analyses were run separately for total crimes and for each crime group.

Finally, we employed the Geographical and Temporal Weighted Regression (GTWR) model to incorporate temporal effects into the analysis. GTWR extends geographically weighted regression by allowing the regression coefficients to vary across both space and time, so that associations between crime and mental health resources can differ by district and year.

Access to mental health resources is not confined to administrative boundaries, and spatial proximity may shape how crimes and services are related across districts. The GTWR model addresses this by applying a spatial kernel function.^37^ Rather than treating each district as an isolated unit, GTWR estimates location-specific coefficients using information from nearby districts, with observations weighted by the distance between district centroids. In this way, relationships between crime and mental health resources in a focal district are informed by patterns in surrounding areas. The bandwidth, which determines the spatial extent of this local influence, was selected using cross-validation to identify an appropriate scale of spatial interaction.^38^ GTWR analyses were conducted using the GWmodel package in R V.4.3.2. An overview of the analytical workflow is provided in online supplemental figure S1.

To assess spatial and temporal heterogeneity in the estimated associations, we examined the distribution of local GTWR coefficients. The detailed summaries are reported in online supplemental tables S2–S5. Additional methodological details on emerging hot spot classification and GTWR are provided in online supplemental appendix S2.1 and S2.2. As a further robustness check, we estimated supplementary ordinary least squares (OLS) spatial lag models to distinguish local resource effects from spillover effects linked to neighbouring districts. Detailed results are reported in online supplemental appendix S3.1–S3.3, and in online supplemental figures S3–S8.

### Patient and public involvement

Patients or the public were not involved in the design, conduct, reporting or dissemination plans of this research, as the study is based on the analysis of publicly available data, including legal judgements, crime statistics and administrative records.

## Results

### Criminal cases involving mental illness by year and crime types

Given the significance of Criminal Code Article 19 in cases related to mental illness, we identified such judgements by searching for ‘刑法第19條 (Criminal Code Article 19)’, ‘刑法第十九條 (Criminal Code Article 19 with 19 in Traditional Chinese)’ or ‘刑法第１９條 (Criminal Code Article 19 with 19 in full character width)’ within the judgement texts. This search produced 23 390 judgements identified in the initial screening step. After restricting the sample to judgements adjudicated by district courts or the Supreme Court and consolidating repeated proceedings using patterns such as ‘Disobey (不服) {JID} …。’, we identified 6302 unique Article 19-related judgements. This dataset was used to estimate the proportion of criminal cases involving indications of mental illness, as Article 19 serves as the legal basis for determining diminished responsibility.

**Figure 1:**
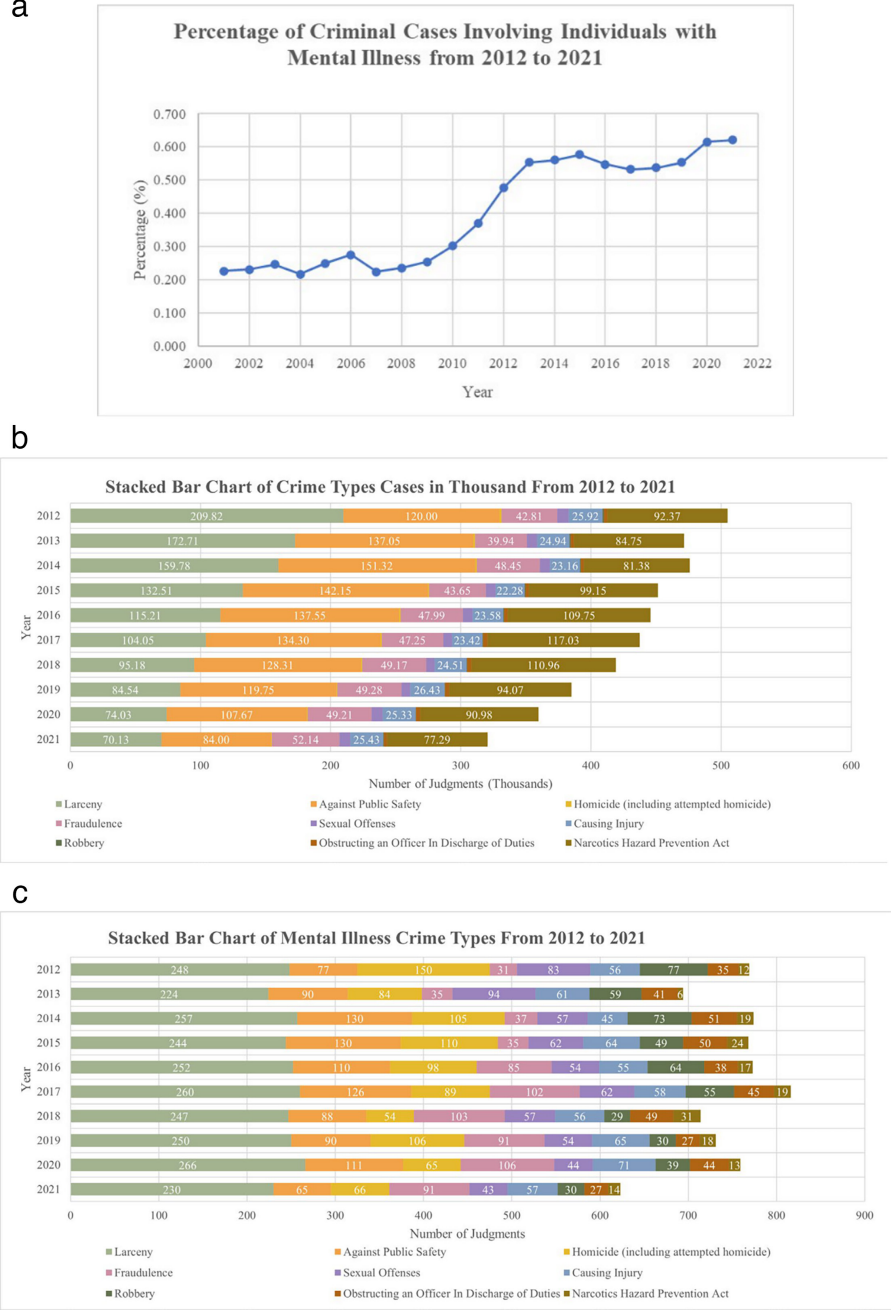
Crime cases with mental illness by year and types. (**a**) Trend of proportion of crime cases involving individuals with mental illness. (**b**) Stacked bar chart of crime types cases in thousand over the years. (**c**) Stacked bar chart of mental illness-related crimes among selected crime types over the years.

Figure 1a illustrates the trend in criminal cases involving individuals with mental illness. The overall proportion of Article 19-related judgements among all criminal cases during the study period was about 0.4%. From 2007 to 2013, this percentage increased from 0.22% (787 of 352 047 judgements) to 0.55% (1460 of 263 949 judgements), representing approximately a 2.5-fold increase. A Cochran-Armitage test for trend for these years confirmed a statistically significant upward trend in the proportion of Article 19-related cases Z = 27.93, p<2.2 × 10^―16^. The timing of this increase coincides with the implementation of the revised Criminal Code Article 19, which replaced the vague concepts of ‘insanity’ and ‘diminished mental capacity’ with a combined medical-legal standard based on the presence of a mental disorder or cognitive impairment and the resulting inability to recognise or control unlawful behaviour. After 2013, the proportion of Article 19-related cases plateaued through 2021, reflecting initial frequent use of the revised rule followed by more cautious application as courts and experts reassessed its scope.

Since data on mental health resources were available only from 2012 onward, and because spatial analyses require coverage of all major crime types rather than only Article 19 cases to compute case proportions, we constructed a separate dataset for spatial and GTWR analyses. For this purpose, we compiled all judgements from 2012 to 2021 belonging to the ten most common crime types in the corpus, selected based on their overall frequency to ensure adequate representation across regions. After removing duplicates, 15 350 judgements were retained from an initial pool of 16 132 cases.

To facilitate descriptive comparisons across crime types, we further refined this dataset. Because the subsequent text analysis required access to the FACT (facts of the case; 事實) section of the judgement, we next identified which of these 15 350 cases contained a clearly extractable FACT description using the pattern ‘FACT (事實) {Fact Description} 。REASON (理由)’. From the 15 350 judgements, 8751 cases were selected for descriptive analysis. After removing formatting-related duplicates, 8701 cases remained eligible for this extraction step, and we successfully retrieved FACT descriptions for 8658 of them (99%). These 8658 cases form the basis for the descriptive comparisons shown in figure 1b and c.

Figure 1b shows crime types in thousands from 2012 to 2021, reflecting a decrease in selected crime types. In contrast, figure 1c depicts the relatively small number of mental illness-related cases among the selected crime types over the same period. Notable differences between these figures include a decline in larceny cases for total crimes but not among mental illness-related cases, an increase in public safety cases and a rise in fraud cases in 2016, which might be linked to the use of Criminal Code Article 19 for acquittals. Homicide, robbery and obstructing an officer cases were more prevalent among mental illness-related cases, while narcotics cases were less common for individuals with mental illness. Corresponding yearly percentages by crime type are reported in online supplemental table S1. We also analysed word usage in legal judgements from 2012 to 2021 and presented the results in online supplemental figures S2–S4.

### Spatiotemporal analysis

From the 8658 judgements with retrievable FACT descriptions, we next identified which cases contained usable geographic information for spatial and GTWR analyses. Geographic locations were extracted from the reported police office, following patterns such as ‘the sue is reported from (訴由) {Police Office} …。’ or ‘the case is reported from (案經) {Police Office} …。’. After matching short and full police office names and conducting manual checks for duplicated names, police office information was successfully extracted for 5827 judgements (67%). These cases were used for the emerging hot spot analysis and for constructing the spatial panels required for GTWR.

Because crime categories differed substantially in scale and distribution, we grouped the 10 crime types into four analytical categories: larceny, narcotics hazard prevention act, violent crime (including homicide, attempted homicide, sexual offences, causing injury and robbery) and normal crime (including obstructing an officer in discharge of duties, against public safety and fraudulence).

**Figure 2:**
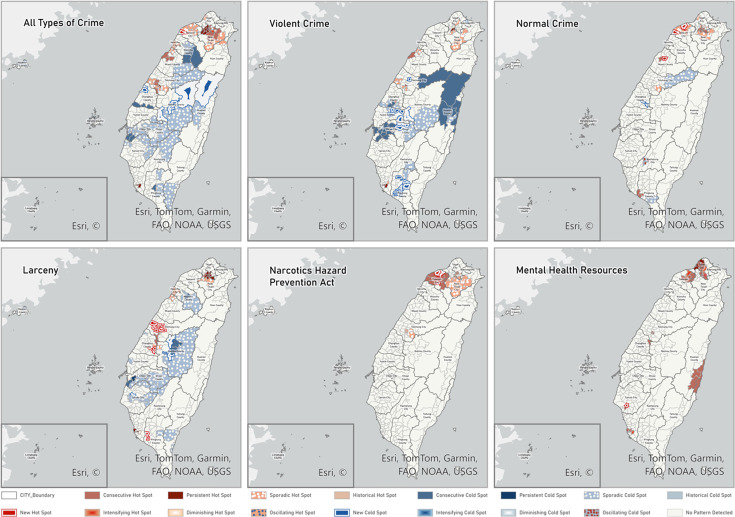
Emerging hot spot maps for number of crimes involving individuals with mental illness and mental health resources. A summary measure of mental health resources is calculated using the following equation: Number of beds + 2 × Number of psychologists and social workers + 3 × Number of doctors + 4 × Number of psychiatry departments. FAO, Food and Agriculture Organization; NOAA, National Oceanic and Atmospheric Administration; USGS, United States Geological Survey. Esri, TomTom, and Garmin are retained as company names, as shown in the figure artwork.

The emerging hot spot map in figure 2 shows the trends of intensifying, diminishing or continuing hot and cold spots in Taiwan, highlighting a prominent hot spot in northern Taiwan, particularly south of New Taipei and near Taoyuan. These clusters spatially overlap with areas of higher population density and major transport hubs, including Taoyuan International Airport, which is a key entry point for narcotics. For other crime types, areas in central Taiwan tend to appear as cold spots, likely reflecting the low population density around the Central Mountain Range. At the same time, emerging hot spots for mental health resources are concentrated in northern Taiwan and a few eastern administrative regions.

**Figure 3:**
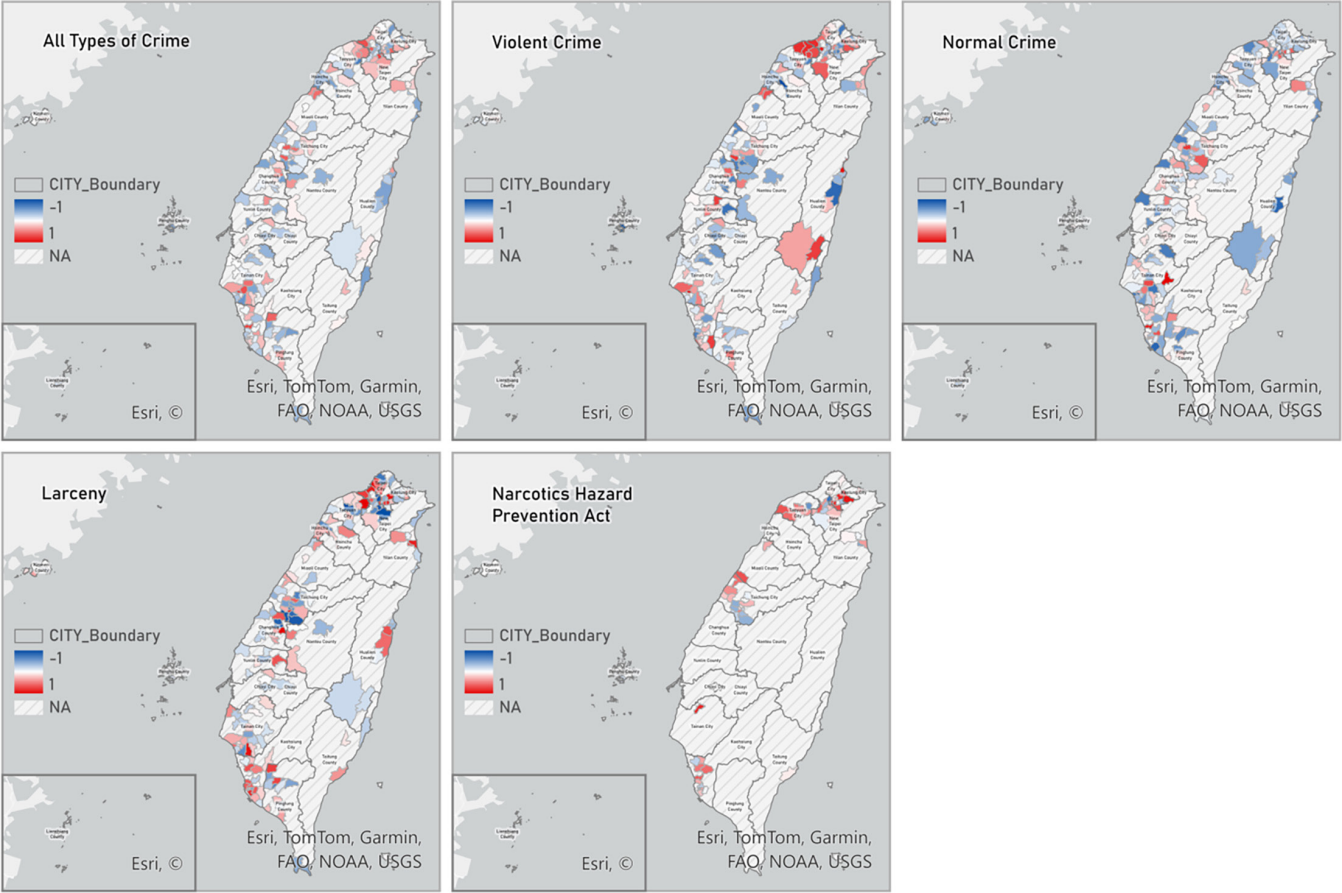
Correlations between mental health resources and number of crimes with individuals involving mental illness. A summary measure of mental health resources is calculated using the following equation: Number of beds + 2 × Number of psychologists and social workers + 3 × Number of doctors + 4 × Number of psychiatry departments.

Figure 3 displays the spatial distribution of correlations between the summary index of mental health resources (Number of beds + 2 × Number of psychologists and social workers + 3 × Number of doctors + 4 × Number of psychiatry departments) and the number of crimes involving individuals with mental illness for each crime category. Districts where either variable showed little to no variation across the study period were not included in the correlation calculation and are displayed as NA in figure 3.

Among districts with non-NA values in figure 3, northwestern Taiwan tends to show more positive bivariate correlations between mental health resources and violent crimes or larceny, whereas many central and eastern districts display weaker or negative correlations, particularly for normal crimes. These map-based patterns are descriptive and exploratory, visualising the geographic distribution of simple correlations and highlighting where stronger or weaker associations tend to occur. However, these correlations do not account for spatial or temporal dependence, differences in population scale across districts or other covariates and therefore are not used as the primary basis for statistical inference. The magnitude and direction of associations between mental health resources and crime are instead evaluated using the GTWR models reported below.

To further examine the relationships between different mental health resources and types of crimes over time, table 1 presents the GTWR estimates for the mental illness crime rate (crimes involving individuals with mental illness per 10 000 people) and the mental illness case proportion (crimes involving individuals with mental illness per 100 cases). The key difference between two analyses is that crime rate considers the entire population as potential offenders, while case proportion focuses solely on those who have already committed crimes. The coefficients reported in tables 1 and 2 summarise the average GTWR estimates across districts and years, with 95% CIs, and are used to describe the central tendency of the underlying spatiotemporal relationships.

**Table 1:** GTWR models using mental health resources to predict crime with mental illness per 10 000 people and mental illness case proportion

Dependent variable	Crime rate (crime with mental illness per 10 000 people)				
	All	Violent	Normal	Larceny	Drug
Predictors	*Estimates* (*CI*)	*Estimates* (*CI*)	*Estimates* (*CI*)	*Estimates* (*CI*)	*Estimates* (*CI*)
Year (year 2012=0)	0	−0.001***	0	0	0
	(−0.001 to 0.000)	(−0.001 to −0.000)	(−0.000 to 0.000)	(−0.000 to 0.000)	(−0.000 to 0.000)
Clinical psychologist (clinic)	2.918*	−0.211	1.158	1.957***	0.014
	(0.616 to 5.220)	(−1.230 to 0.807)	(−0.116 to 2.433)	(1.110 to 2.804)	(−0.210 to 0.239)
Counselling psychologist (clinic)	−1.361	−0.156	−0.521	−0.670*	−0.014
	(−2.889 to 0.167)	(−0.831 to 0.520)	(−1.367 to 0.324)	(−1.232 to −0.108)	(−0.163 to 0.135)
Clinical social worker (clinic)	3.217***	1.527***	0.042	1.676***	−0.029
	(1.749 to 4.684)	(0.878 to 2.177)	(−0.771 to 0.854)	(1.137 to 2.216)	(−0.172 to 0.114)
Psychiatry (clinic)	−0.409	0.021	−0.294	−0.189	0.054
	(−1.309 to 0.491)	(−0.378 to 0.419)	(−0.792 to 0.204)	(−0.520 to 0.142)	(−0.034 to 0.141)
Clinical psychologist (hospital)	0.08	0.111	−0.072	0.028	0.013
	(−0.405 to 0.566)	(−0.104 to 0.326)	(−0.341 to 0.196)	(−0.151 to 0.206)	(−0.034 to 0.061)
Counselling psychologist (hospital)	−1.58	−0.331	−0.741	−0.449	−0.059
	(−3.197 to 0.037)	(−1.046 to 0.384)	(−1.636 to 0.154)	(−1.044 to 0.146)	(−0.216 to 0.099)
Clinical social worker (hospital)	0.056	0.01	−0.014	0.052	0.008
	(−0.130 to 0.242)	(−0.072 to 0.092)	(−0.117 to 0.088)	(−0.016 to 0.121)	(−0.010 to 0.026)
Psychiatric acute bed	0.003	0.012	−0.015	0.005	0
	(−0.026 to 0.032)	(−0.000 to 0.025)	(−0.031 to 0.001)	(−0.005 to 0.016)	(−0.002 to 0.003)
Psychiatric chronic bed	0.001	0	0	0.001	0
	(−0.006 to 0.008)	(−0.003 to 0.003)	(−0.003 to 0.004)	(−0.001 to 0.004)	(−0.001 to 0.000)
Psychiatric intensive care bed	−0.331	−0.255*	0.017	−0.078	−0.015
	(−0.879 to 0.217)	(−0.497 to −0.012)	(−0.287 to 0.320)	(−0.279 to 0.124)	(−0.069 to 0.038)
Psychiatry (hospital)	2.785***	−0.043	2.511***	0.243	0.073
	(1.840 to 3.729)	(−0.460 to 0.375)	(1.988 to 3.034)	(−0.104 to 0.591)	(−0.019 to 0.165)
Psychiatry doctor	−0.172	−0.054	−0.011	−0.093*	−0.015
	(−0.377 to 0.032)	(−0.144 to 0.037)	(−0.124 to 0.103)	(−0.169 to −0.018)	(−0.035 to 0.005)
Low-income household	0.002***	0.001***	0.001***	0.001***	0
	(0.002 to 0.003)	(0.000 to 0.001)	(0.000 to 0.001)	(0.001 to 0.001)	(−0.000 to 0.000)
College degree	0	0.000*	0	0	0
	(−0.000 to 0.001)	(0.000 to 0.000)	(−0.000 to 0.000)	(−0.000 to 0.000)	(−0.000 to 0.000)
Intercept	0.046*	0.059***	−0.013	−0.001	0.001
	(0.004 to 0.087)	(0.041 to 0.078)	(−0.036 to 0.010)	(−0.017 to 0.014)	(−0.003 to 0.005)
Observations	3650	3650	3650	3650	3650
Adjusted R^2^	0.032	0.022	0.028	0.036	0.002

Dependent variable	Mental illness case proportion (crime with mental illness per 100 cases)				
	All	Violent	Normal	Larceny	Drug
Predictors	*Estimates* (*CI*)	*Estimates* (*CI*)	*Estimates* (*CI*)	*Estimates* (*CI*)	*Estimates* (*CI*)
Year (year 2012=0)	0	−0.009***	0	0.003***	0
	(−0.001 to 0.000)	(−0.011 to −0.006)	(−0.001 to 0.001)	(0.002 to 0.004)	(−0.000 to 0.000)
Clinical psychologist (clinic)	4.396*	−5.731	2.279	13.334***	−0.024
	(0.924 to 7.869)	(−20.404 to 8.943)	(−1.968 to 6.525)	(7.015 to 19.652)	(−1.634 to 1.586)
Counselling psychologist (clinic)	−1.634	−3.986	−0.576	−5.689**	−0.004
	(−3.939 to 0.670)	(−13.723 to 5.751)	(−3.394 to 2.242)	(−9.882 to −1.496)	(−1.073 to 1.064)
Clinical social worker (clinic)	8.557***	29.401***	1.02	25.898***	−0.172
	(6.343 to 10.771)	(20.046 to 38.756)	(−1.687 to 3.728)	(21.870 to 29.927)	(−1.198 to 0.855)
Psychiatry (clinic)	−1.447*	−1.56	−1.208	−2.978*	0.177
	(−2.804 to −0.089)	(−7.297 to 4.177)	(−2.868 to 0.453)	(−5.448 to −0.507)	(−0.453 to 0.807)
Clinical psychologist (hospital)	−0.236	−1.087	−0.432	−0.875	−0.06
	(−0.968 to 0.496)	(−4.181 to 2.008)	(−1.328 to 0.463)	(−2.208 to 0.457)	(−0.400 to 0.279)
Counselling psychologist (hospital)	−2.364	−4.761	−2.261	−3.215	−0.167
	(−4.803 to 0.074)	(−15.066 to 5.544)	(−5.244 to 0.721)	(−7.652 to 1.223)	(−1.298 to 0.964)
Clinical social worker (hospital)	−0.01	0.665	−0.122	0.488	0.055
	(−0.290 to 0.270)	(−0.519 to 1.849)	(−0.465 to 0.221)	(−0.021 to 0.998)	(−0.075 to 0.185)
Psychiatric acute bed	−0.008	0.239*	−0.057*	0.051	0.002
	(−0.051 to 0.035)	(0.056 to 0.422)	(−0.110 to −0.004)	(−0.027 to 0.130)	(−0.018 to 0.022)
Psychiatric chronic bed	0.004	0.01	0	0.016	0
	(−0.007 to 0.015)	(−0.036 to 0.055)	(−0.013 to 0.013)	(−0.004 to 0.036)	(−0.005 to 0.005)
Psychiatric intensive care bed	−0.053	−3.558*	0.304	−0.125	−0.073
	(−0.880 to 0.774)	(−7.053 to −0.063)	(−0.707 to 1.316)	(−1.630 to 1.380)	(−0.456 to 0.311)
Psychiatry (hospital)	5.350***	−4.879	9.243***	0.53	0.383
	(3.925 to 6.774)	(−10.899 to 1.142)	(7.501 to 10.986)	(−2.062 to 3.123)	(−0.277 to 1.044)
Psychiatry doctor	−0.299	−0.585	−0.055	−0.632*	−0.059
	(−0.608 to 0.010)	(−1.890 to 0.721)	(−0.433 to 0.323)	(−1.195 to −0.070)	(−0.203 to 0.084)
Low-income household	0.002***	0.005*	0.001	0.006***	0
	(0.001 to 0.003)	(0.001 to 0.009)	(−0.001 to 0.002)	(0.004 to 0.008)	(−0.000 to 0.001)
College degree	0.001***	0.004***	0.001*	0.001**	0
	(0.001 to 0.001)	(0.002 to 0.006)	(0.000 to 0.001)	(0.000 to 0.002)	(−0.000 to 0.000)
Intercept	0.05	0.935***	0.007	−0.343***	−0.005
	(−0.013 to 0.113)	(0.669 to 1.201)	(−0.070 to 0.084)	(−0.457 to −0.228)	(−0.034 to 0.025)
Observations	3650	3650	3650	3650	3650
Adjusted R^2^	0.038	0.023	0.030	0.069	−0.001

Models were estimated using a Geographically and Temporally Weighted Regression (GTWR) framework to allow regression coefficients to vary across districts and years. The coefficients reported in this table summarise the average GTWR estimates across space and time and are presented with 95% CI for ease of interpretation. Distributions of local GTWR coefficients, including the median, 25th percentile (Q25) and 75th percentile (Q75), are reported in online supplemental tables S2–S5. *p<0.05; **p<0.01; ***p<0.001.

**Table 2:** GTWR models using mental health resource summary to predict crime with mental illness per 10 000 people and mental illness case proportion

Dependent variable	Crime rate (crime with mental illness per 10 000 people)				
	All	Violent	Normal	Larceny	Drug
Predictors	*Estimates* (*CI*)	*Estimates* (*CI*)	*Estimates* (*CI*)	*Estimates* (*CI*)	*Estimates* (*CI*)
Year (year 2012=0)	0	−0.001***	0	0	0
	(−0.001 to 0.000)	(−0.001 to −0.000)	(−0.000 to 0.000)	(−0.000 to 0.000)	(−0.000 to 0.000)
Psychologist and social worker	0.125*	0.045	0.013	0.058**	0.009
	(0.023 to 0.227)	(−0.000 to 0.090)	(−0.044 to 0.069)	(0.021 to 0.096)	(−0.001 to 0.019)
Psychiatry	0.975**	−0.071	0.974***	0.012	0.06
	(0.354 to 1.596)	(−0.345 to 0.203)	(0.630 to 1.319)	(−0.217 to 0.241)	(−0.000 to 0.120)
Psychiatric bed	0.005	0.002	0.001	0.002	0
	(−0.000 to 0.010)	(−0.000 to 0.004)	(−0.002 to 0.004)	(−0.000 to 0.004)	(−0.001 to 0.000)
Psychiatry doctor	−0.261**	−0.059	−0.088	−0.099**	−0.015
	(−0.455 to −0.066)	(−0.145 to 0.026)	(−0.196 to 0.020)	(−0.170 to −0.027)	(−0.034 to 0.004)
Low-income family	0.002***	0.001***	0.001**	0.001***	0
	(0.001 to 0.003)	(0.000 to 0.001)	(0.000 to 0.001)	(0.001 to 0.001)	(−0.000 to 0.000)
College degree	0	0.000*	0	0	0
	(−0.000 to 0.000)	(0.000 to 0.000)	(−0.000 to 0.000)	(−0.000 to 0.000)	(−0.000 to 0.000)
Intercept	0.051*	0.060***	−0.011	0.001	0.001
	(0.010 to 0.093)	(0.042 to 0.079)	(−0.034 to 0.012)	(−0.014 to 0.017)	(−0.003 to 0.005)
Observations	3650	3650	3650	3650	3650
Adjusted R^2^	0.020	0.016	0.013	0.022	0.003

Dependent variable	Mental illness case proportion (crime with mental illness per 100 cases)				
	All	Violent	Normal	Larceny	Drug
Predictors	*Estimates* (*CI*)	*Estimates* (*CI*)	*Estimates* (*CI*)	*Estimates* (*CI*)	*Estimates* (*CI*)
Year (year 2012=0)	−0.001	−0.009***	0	0.003***	0
	(−0.001 to 0.000)	(−0.011 to −0.006)	(−0.001 to 0.001)	(0.002 to 0.004)	(−0.000 to 0.000)
Psychologist and social worker	0.085	0.423	−0.026	0.322*	0.031
	(−0.071 to 0.240)	(−0.228 to 1.074)	(−0.215 to 0.163)	(0.037 to 0.608)	(−0.040 to 0.102)
Psychiatry	1.566**	−3.964*	3.560***	−1.529	0.271
	(0.622 to 2.511)	(−7.921 to −0.007)	(2.410 to 4.709)	(−3.263 to 0.205)	(−0.160 to 0.702)
Psychiatric bed	0.008	0.031	0.003	0.015	0
	(−0.000 to 0.016)	(−0.004 to 0.066)	(−0.008 to 0.013)	(−0.001 to 0.030)	(−0.004 to 0.004)
Psychiatry doctor	−0.504***	−0.877	−0.348	−0.837**	−0.072
	(−0.799 to −0.209)	(−2.115 to 0.360)	(−0.708 to 0.012)	(−1.379 to −0.294)	(−0.207 to 0.063)
Low-income family	0.002***	0.005*	0	0.006***	0
	(0.001 to 0.003)	(0.001 to 0.009)	(−0.001 to 0.001)	(0.004 to 0.008)	(−0.000 to 0.001)
College degree	0.001***	0.004***	0	0.002***	0
	(0.000 to 0.001)	(0.002 to 0.006)	(−0.000 to 0.001)	(0.001 to 0.002)	(−0.000 to 0.000)
Intercept	0.062	0.966***	0.015	−0.313***	−0.004
	(−0.002 to 0.125)	(0.701 to 1.232)	(−0.062 to 0.092)	(−0.430 to −0.197)	(−0.033 to 0.025)
Observations	3650	3650	3650	3650	3650
Adjusted R^2^	0.011	0.013	0.012	0.026	0.001

Models were estimated using a Geographically and Temporally Weighted Regression (GTWR) framework to allow regression coefficients to vary across districts and years. The coefficients reported in this table summarise the average GTWR estimates across space and time and are presented with 95% CIs for ease of interpretation. Distributions of local GTWR coefficients, including the median, 25th percentile (Q25) and 75th percentile (Q75), are reported in online supplemental tables S2–S5. *p<0.05; **p<0.01; ***p<0.001.

Several findings related to mental health resources emerged from the analysis (table 1). First, psychologists and social workers in clinics appeared to have a stronger influence on the mental illness crime rate and case proportion than their counterparts in hospitals. For example, clinic-based clinical social workers were positively associated with the overall mental illness crime rate (β=3.217, 95% CI 1.749 to 4.684) and the overall mental illness case proportion (β=8.557, CI 6.343 to 10.771), whereas the corresponding hospital-based estimates were small and not statistically distinguishable from zero. This positive association may partly reflect post-incident identification and recording of mental illness following criminal events, particularly for clinic-based psychologists and social workers. Second, psychiatry departments in hospitals generally correlated positively with crime rates, including overall crime rate (β=2.785, CI 1.840 to 3.729) and normal crime rate (β=2.511, CI 1.988 to 3.034), while psychiatry clinics showed negative correlations with case proportions, including overall case proportion (β = −1.447, CI −2.804 to −0.089) and larceny case proportion (β = −2.978, CI −5.448 to −0.507). This implies that an increase in psychiatry services per 100 people could potentially reduce the number of criminal cases or larceny cases involving individuals with mental illness. Third, an increased number of psychiatric intensive care beds was associated with reductions in violent crime outcomes, including violent crime rate (β = −0.255, CI −0.497 to −0.012) and violent case proportion (β = −3.558, CI −7.053 to −0.063). Finally, the number of psychiatry doctors negatively correlated with all crime rates and larceny rates, most notably for larceny crime rate (β = −0.093, CI −0.169 to −0.018) and larceny case proportion (β = −0.632, CI −1.195 to −0.070).

Summarising mental health resources into broader categories, table 2 presents the associations between these summary indices and crime rates or case proportions. The density of psychologists and social workers tended to show a positive relationship with crimes, including overall crime rate (β=0.125, CI 0.023 to 0.227) and larceny crime rate (β=0.058, CI 0.021 to 0.096). In contrast, while the density of psychiatry departments had positive relationships with all crimes and normal crimes, including overall crime rate (β=0.975, CI 0.354 to 1.596) and normal crime rate (β=0.974, CI 0.630 to 1.319), it negatively correlated with violent crimes, especially in the case proportion analysis (β = −3.964, CI −7.921 to −0.007). Furthermore, the density of psychiatry doctors consistently showed a negative relationship with all types of crime, particularly when the outcome was crime rate, including overall crime rate (β = −0.261, CI −0.455 to −0.066) and larceny crime rate (β = −0.099, CI −0.170 to −0.027).

In addition to these findings on mental health resources, we observed that the number of low-income households was positively associated with crime rates but not case proportions. For example, low-income households showed a positive association with overall mental illness crime rate (β=0.002, CI 0.002 to 0.003) and with violent crime rate (β=0.001, CI 0.000 to 0.001), while the corresponding associations with case proportions were smaller and in several models not statistically distinguishable from zero. In contrast, the proportion of the population with a college degree showed a positive association with case proportions but not crime rates, including overall case proportion (β=0.001, CI 0.001 to 0.001) and violent case proportion (β=0.004, CI 0.002 to 0.006). Although these effects were modest in magnitude, they suggest that poverty is linked to mental illness-related crimes in the general population, while higher education levels among residents may be associated with mental illness among offenders.

To assess spatial and temporal heterogeneity in the estimated associations, we examined the distribution of local GTWR coefficients. Detailed summaries are reported in online supplemental tables S2–S5.

## Discussion

Based on information recorded in legal judgements, we estimate that about 0.4% of criminal cases from 2000 to 2022 involved individuals with indications of mental illness. To our knowledge, this is the first study using NLP to analyse legal judgement to estimate the number of crimes involving individuals with mental illness. Among the different types of analysis, 9.92% of homicide cases and 8.47% of robbery cases involved individuals with mental illness, while the percentage ranges from 0.02% (drugs) to 0.82% (sexual offences) for the other types of crimes from 2012 to 2021. Given the number of homicide and robbery cases is relatively rare in Taiwan, the observed percentages of cases involving individuals with mental illness are low. Our findings align with previous studies showing that mental disorders account for only a small number of crimes. Prior research has reported that only 1–5% of violent incidents can be attributed solely to mental illness, that about 7.5% of crimes among offenders with serious mental illness are directly related to psychiatric symptoms, and that mentally abnormal homicides account for roughly 3–4% of all homicide cases.^7^,^11^ While the number of Article 19-related cases increased in the early 2010s, with the proportion of such judgements among all criminal cases rising from about 0.22% in 2007 to about 0.55% in 2013, this rise was followed by a plateau through 2021. This pattern is consistent with an initial phase of growing awareness and wider adoption of Criminal Code Article 19, which allows reduced sentences in cases involving diminished responsibility, followed by more cautious and stable use as courts and the public debated its appropriate scope. Together with the low overall proportion of Article 19-related cases, this plateau supports the conclusion that the relationship between mental illness and crime remains relatively weak at the population level.

In addition to estimating the number of crimes related to mental illness, we used the geographic locations of police stations reported in judgements to identify emerging hot and cold spots of crimes involving mental illness. We linked these locations to statistics on mental health resources in the same areas to explore their relationship with crimes involving mental illness. Emerging hot spots of such crimes were positively correlated with hot spots of mental health resources, particularly for violent crime and larceny. While causal relationships could not be established due to data limitations, we speculate several possible explanations for these positive correlations. One explanation was that victims of violence and residents near crime hot spots may require mental health resources to cope with heightened risks of post-traumatic stress disorder (PTSD) and depression.^39^ Another possibility was that both mental health resources and crime rates are concentrated in urban areas, a common pattern in East Asian countries.^40 41^

The association between mental health resources and crimes involving mental illness was negative in the case of normal crime. The GTWR model further revealed a negative relationship between the density of psychiatry doctors and all types of crime, especially larceny, while the density of psychiatry departments was negatively associated with violent crimes. Although some mental health resources were positively correlated with crimes involving mental illness, possibly due to urban concentration and residents seeking coping mechanisms, the negative associations suggested that increasing resources like psychiatry departments and doctors could help reduce certain crimes. In Taiwan, only psychiatry doctors can prescribe medications for mental disorders, which may explain why our findings imply that psychotropic drug use^20^ and mental healthcare services covered by universal health insurance^21 22^ could be effective tools for addressing crimes involving mental illness.

The GTWR results also point to meaningful spatial and temporal heterogeneity in the associations between mental health resources and crime. Although the global GTWR estimates reported in tables 1 and 2 capture the central tendency of these relationships, the distribution of local coefficients shows that the effects of mental health resources are not uniform across Taiwan. In particular, clinic-based mental health personnel and psychiatry-related resources exhibit wide IQRs for violent crime and larceny, indicating that the strength of these associations varies across regions and over time. Such variations likely reflect differences in local service accessibility, case composition and institutional practices related to reporting and referral. These findings suggest that conclusions based solely on average effects may mask important local dynamics, and they highlight the value of spatiotemporal approaches for understanding how mental health infrastructure relates to crime in different contexts.

Our study examined the relationship between mental illness, mental health resources and crime using the text of legal judgements. Legal responsibility and sentence mitigation in cases involving mental illness are subjects of ongoing legal and societal debate worldwide. In the United States, for example, preventive detention based on mental disorders is generally viewed as requiring careful restraint to protect individual liberty, and individuals may be found not guilty by reason of insanity or receive mitigated sentences when mental illness substantially impairs criminal responsibility.^42 43^ At the same time, public perceptions increasingly associate mental illness with dangerousness, contributing to support for legally mandated treatment in severe cases.^3^

Similarly, in Taiwan, Criminal Code Article 19 permits reduced sentences for individuals whose mental disorders significantly impair their ability to understand or control their actions, sparking public debate over fairness and public safety. This debate is further complicated by stigma, often perpetuated by media portrayals linking mental illness to violent crime. Research highlights the societal implications of such stigma and emphasises the importance of destigmatisation.^44^ Public concerns about Article 19 in Taiwan often centre on scepticism towards forensic evaluations, particularly when they result in reduced sentences, sparking demands for stricter risk management and effective containment rather than leniency.^34^ While these issues are beyond the scope of this study, by demonstrating that criminal cases involving mental illness account for a small proportion of total crimes and showing that mental health resources can help reduce these types of crimes, we hope to contribute to the destigmatisation of individuals with mental illness and advocate for equitable treatment in justice systems worldwide.

### Study strengths and limitations

This study integrates large-scale legal judgement data with administrative records on mental health resources and applies NLP and spatial methods to examine spatiotemporal patterns of crimes involving individuals with mental illness. However, several limitations should be noted. Legal judgements are not written in a standardised format, which may affect the accuracy of automated text extraction, and geographic locations inferred from police office names may not precisely reflect incident locations. In addition, the use of area-level administrative data raises the possibility of ecological inference, and the cross-sectional nature of the data limits causal interpretation. Despite these limitations, the consistency of results across multiple outcomes and robustness checks supports the validity of the main findings.

Future work could extend this approach in several ways. Continued development of NLP tools tailored to legal texts would improve identification of mental illness in judgements. Access to finer-grained spatial data and, where feasible, linkage between legal and mental health service records would allow more direct evaluation of the proxy indicators used in this study. Comparative analyses across jurisdictions may further clarify how legal and clinical institutions shape criminal justice outcomes.

## Conclusion

Our study used NLP and GIS to examine the relationship between mental illness, mental health resources and crime in Taiwan. We found that a small fraction of crimes involved individuals with mental disorders, consistent with global findings on the limited link between mental illness and criminal behaviour. Additionally, areas with greater availability of mental health resources often corresponded to higher crime rates for certain offences, likely reflecting urbanisation and the need for victim support services. Importantly, a higher density of psychiatry departments and doctors was associated with reductions in some crimes involving mental illness, highlighting the potential benefits of increasing targeted mental health resources. These findings underscore the importance of expanding access to mental healthcare and addressing the stigma surrounding mental illness.

## Supplementary material

10.1136/bmjph-2025-002832 online supplemental file 1

## Data Availability

Data are available in a public, open access repository.
