# Supplementary material for: Mental illness, crime and mental health resources: insights from Taiwan using text analysis and spatio-temporal analysis
Source: BMJ Public Health. 2026 May 6;4(2):e002832. doi: 10.1136/bmjph-2025-002832 (PMC13150923; doi:10.1136/bmjph-2025-002832)
Supplement: online supplemental file 1 [file bmjph-4-2-s001.docx]

Supplementary Appendix S1. Additional Methods for Legal Text Analysis

This appendix provides additional technical details related to the identification of mental illness in legal judgments.

S1.1. Legal Context of Criminal Code Article 19

The current wording of Article 19 was introduced in a 2005–2006 revision that replaced the earlier terms “insanity” and “diminished mental capacity” (心神喪失 and 精神耗弱) with a combined medical–legal standard. Under the revised provision [1], exemptions or reductions in criminal responsibility require both the presence of a mental disorder or other cognitive impairment and a resulting inability, or markedly reduced ability, to recognize the unlawfulness of one’s conduct or to act in accordance with such recognition. This change was intended to move away from vague, discretionary impressions of “mental abnormality” and to anchor decisions more firmly in structured forensic psychiatric evaluations. In practice, it has reduced the scope for courts to characterize a defendant as “not mentally ill” based purely on their own impressions and has made formal psychiatric assessment a central part of determining criminal responsibility.

S1.2. Limitations of Keyword-Based Identification in Chinese Legal Texts

In practice, because legal judgments in Chinese are written as continuous text without word boundaries, simple keyword searches can lead to segmentation ambiguity. For example, characters associated with clinical diagnoses may also appear in unrelated idioms or descriptive phrases. This issue is well documented in research on Chinese natural language processing [2]. To reduce the risk of false matches and ensure that extracted terms reflected meaningful legal or clinical context, we applied CkipTagger, a neural network-based word-segmentation and part-of-speech tagging model [3], before identifying terms near Article 19.

S1.3. NLP Preprocessing Using CkipTagger

This study uses CkipTagger to segment words in legal judgments. CkipTagger, developed by the CKIP (Chinese Knowledge and Information Processing) group at Academia Sinica [3], is an open-source library for Chinese natural language processing (NLP). It implements the WS-POS-NER pipeline for word segmentation, part-of-speech tagging, and named entity recognition, using biLSTM techniques for enhanced performance. CkipTagger achieves high precision, recall, and F1-score for word segmentation (97.49%, 97.19%, and 97.33%, respectively) and maintains 94.59% accuracy for part-of-speech tagging. Compared to classic tools like CKIPWS and Jieba-zh_TW, CkipTagger consistently delivers superior results. Additionally, it incorporates named entity recognition (NER), a feature absent in earlier tools.

Processing Chinese legal documents requires word segmentation because, unlike English, Chinese text is written without whitespace. This characteristic makes simple keyword searches prone to segmentation ambiguity. Foundational work in Chinese information retrieval has shown that character-based matching can incorrectly identify unrelated phrases as target terms [2]. For example, a search for "憂鬱(depression)" may also capture "憂鬱的天氣(gloomy weather)," and the character "鬱" appears in unrelated words such as "鬱金香(tulip)." To reduce these errors, we applied CkipTagger’s segmentation and part-of-speech -tagging functions, which build on the neural architectures described by Li et al. (2020) [3]. This approach identifies linguistically valid units and allows us to distinguish clinical diagnoses and legal terminology from incidental narrative expressions in the judgment texts.

In this study, we used CkipTagger to preprocess fact descriptions extracted from judgments, following the pattern "FACT (事實) {Fact Description} 。REASON (理由)". Its robust capabilities in word segmentation, part-of-speech tagging, and named entity recognition enhanced the accuracy and efficiency of text processing for further analysis. Text analysis was performed using Python 3.10.

Supplementary Appendix S2. Spatiotemporal Methods

This appendix provides additional technical details on the spatiotemporal analytical methods used in the study, including the classification of emerging hot and cold spots and the specification and implementation of the Geographical and Temporal Weighted Regression (GTWR) model.

S2.1. Emerging Hot Spot Classification Rules

Based on these spatial and temporal patterns, each location is assigned to one of the emerging hot or cold spot categories according to the default ArcGIS Pro definitions. When the most recent time step is a statistically significant hot spot, a location is classified as a New hot spot if this is the first time a hot spot appears. It is classified as a Consecutive hot spot if there is a single uninterrupted run of hot spot time steps that comprises less than 90% of all intervals. Locations in which at least 90% of the time steps are hot spots and the Gi* values are increasing over time are labeled Intensifying hot spots, whereas those with at least 90% hot spot time steps and no significant trend are labeled Persistent hot spots. When at least 90% of the time steps are hot spots but Gi* values are decreasing over time, the location is categorized as a Diminishing hot spot. Locations with hot spots in some, but not all, time steps without a consistent trend are labeled Sporadic hot spots, and locations that alternate between significant hot and cold spots over time are labeled Oscillating hot spots. If the most recent time step is not a hot spot but at least 90% of the time steps were hot spots, the location is categorized as a Historical hot spot. The same set of rules is applied to cold spots, based on statistically significant low Gi* values and trends in clustering of low values over time.

S2.2. Geographical and Temporal Weighted Regression (GTWR): Model Formulation

Mathematically, the GTWR model can be represented as:

$$Y_{i}=\beta_{0}\left( u_{i}, v_{i}, t_{i} \right)+\sum_{k} \beta_{k}\left( u_{i}, v_{i}, t_{i} \right)X_{ik}+\varepsilon_{i}$$

where $Y_{i}$ is the crime rate in observation $i$(a specific district–year), $X_{ik}$ are the covariates, and $\beta_{k}(u_{i},v_{i},t_{i})$ local regression coefficient for covariate $k$ at space-time location $\left( u_{i},v_{i},t_{i} \right)$. Here, $u_{i}$ and $v_{i}$ are the planar coordinates of the centroid of district $i_{i}$, and $t_{i}$ is the calendar year (2012–2021).

The vector of local coefficients at $\left( u_{i},v_{i},t_{i} \right)$ is estimated as

$$\hat{\beta}\left( u_{i},v_{i},t_{i} \right)=\left[ X^{T}W(u_{i},v_{i},t_{i})X \right]^{-1}X^{T}W(u_{i},v_{i},t_{i})Y$$

where $X$ is the design matrix of covariates, $Y$ is the vector of outcomes, and $W\left( u_{i},v_{i},t_{i} \right)$ is an $n\times n$ diagonal weight matrix whose elements $\alpha_{ij} \left( 1\leq j\leq n \right)$ depend on the spatiotemporal distance between observation $i$ and observation $j$. Nearby observations in space and time receive larger weights, whereas distant observations receive smaller weights.

In practice, we first aggregated crime counts and mental health resource variables to the district (鄉鎮市區)-by-year level, yielding a panel of 365 districts observed from 2012 to 2021. Each district polygon was obtained from the national administrative boundary shapefile, and its centroid was computed using the sf package in R to provide the spatial coordinates $\left( u_{i},v_{i} \right)$. We then calculated Euclidean distances between district centroids and absolute differences in years, and combined these into a single spatiotemporal distance matrix using the st.dist function from the GWmodel package, with a Gaussian kernel to combine spatial distances between district centroids and temporal distances between years into a single spatiotemporal distance matrix. The bandwidth, which determines the extent of this local influence, was selected by cross-validation (bw.gtwr) for each outcome.

Supplementary Appendix S3. Additional Results

This appendix presents supplementary results that support and contextualize the main findings, including textual analyses of legal judgments, robustness checks using spatial lag models, and distributional summaries of local GTWR coefficients.

S3.1. Textual Context of Article 19 in Legal Judgments

We also analyzed word usage in legal judgments from 2012 to 2021. Supplementary Figure S2 summarizes the key terms that appear near Article 19 in the judgment texts. Commonly used terms include "精神 (psychiatric)," "有期徒刑 (fixed term imprisonment)," and "竊盜 (larceny)." Supplementary Figure S3 presents the transition in terminology for schizophrenia in Chinese, showing the change from "分裂症/分裂病 (mind-splitting disorder)" to "思覺失調症 (schizophrenia spectrum disorder)" in 2014, as well as trends in commonly used words such as "mental disorder," "schizophrenia" and "depressive disorder." Notably, the term "psychotic disorder" showed a decreasing trend. Supplementary Figure S4 summarizes the most frequently mentioned locations in the judgments, including Kaohsiung, Taipei, Taichung, and New Taipei, suggesting that more cases occur in larger cities, likely due to higher population density.

S3.2. Spatial Lag Models as Robustness Checks

As a further robustness check, we estimated supplementary ordinary least squares (OLS) models with spatial lag terms to examine whether the observed associations between mental health resources and crime outcomes may be influenced by resource availability in neighboring districts rather than local conditions alone. By including spatially lagged covariates, these models distinguish associations attributable to local resource density from spillover effects linked to adjacent areas.

Across crime types and outcomes, the spatial lag results were broadly consistent with the main GTWR findings. Higher local densities of psychiatry doctors and psychiatry services remained associated with lower crime rates and lower proportions of mental illness–related cases after accounting for neighboring resource levels. In addition, higher levels of psychiatry-related resources in neighboring districts also tended to correlate with lower crime in the focal area, suggesting that the potential benefits of these services are not strictly confined to administrative boundaries.

In contrast, psychologists and social workers more often showed positive local associations with crime outcomes, while their neighboring effects were weaker, inconsistent, or mixed across models. Together, these patterns indicate that the main GTWR results are not driven solely by spillover effects from resource-rich neighboring areas, while still underscoring the relevance of broader neighborhood context in shaping observed associations. Full spatial lag model results are reported in Supplementary Figures S5–S8.

S3.3. Distribution of Local GTWR Coefficients

To examine whether these average associations mask spatial or temporal variation, we further assessed the distribution of local GTWR coefficients across space–time units. Supplementary Tables S2 and S3 report the median, 25th percentile (Q25), and 75th percentile (Q75) of local coefficients for models using disaggregated mental health resources, while Supplementary Tables S4 and S5 present the same summaries for models using aggregated resource indices. Across outcomes and crime types, the median local GTWR coefficients generally align in direction and relative magnitude with the estimates reported in Tables 1 and 2, suggesting that the main results reflect the central tendency of the underlying spatiotemporal relationships rather than being driven by a small number of regions or years. At the same time, the interquartile ranges indicate notable heterogeneity for several predictors, particularly clinic-based psychologists and social workers, psychiatry departments, and psychiatric beds. This variability was more pronounced for violent crime and larceny than for normal crime, indicating that the strength of these associations differs meaningfully across regions and over time, even when the overall direction of the relationship remains consistent.

References

1. Ministry of Justice. Legislative reasons for Article 19 of the Criminal Code [Internet]. Laws and Regulations Retrieving System. Ministry of Justice, Republic of China (Taiwan); 2005 [cited 2025 Dec 13]. Available from: https://mojlaw.moj.gov.tw/LawContentReason.aspx?LSID=FL001424&LawNo=19

2. Foo S, Li H. Chinese word segmentation and its effect on information retrieval. Inf Process Manag. 2004 Jan 1;40(1):161–90.

3. Li PH, Fu TJ, Ma WY. Why Attention? Analyze BiLSTM Deficiency and Its Remedies in the Case of NER. Proc AAAI Conf Artif Intell. 2020 Apr 3;34(05):8236–44.

Supplementary Tables and Figures

Supplementary Table S1. Percentage of Crime Cases Involving Individuals with Mental Illness by Top Ten Crime Types in 2012 to 2021

| Year | Larceny | Against Public Safety | Homicide (including attempted homicide) | Fraudulence | Sexual Offenses | Causing Injury | Robbery | Obstructing an Officer in Discharge of Duties | Narcotics Hazard Prevention Act | Top Ten with Mental Illness Over All Crimes Cases |
| --- | --- | --- | --- | --- | --- | --- | --- | --- | --- | --- |
| 2012 | 0.12% | 0.06% | 9.75% | 0.07% | 0.95% | 0.22% | 6.50% | 1.40% | 0.01% | 0.15% |
| 2013 | 0.13% | 0.07% | 7.26% | 0.09% | 1.23% | 0.24% | 6.05% | 1.78% | 0.01% | 0.15% |
| 2014 | 0.16% | 0.09% | 9.05% | 0.08% | 0.74% | 0.19% | 9.17% | 2.44% | 0.02% | 0.16% |
| 2015 | 0.18% | 0.09% | 10.54% | 0.08% | 0.85% | 0.29% | 6.43% | 1.94% | 0.02% | 0.17% |
| 2016 | 0.22% | 0.08% | 10.54% | 0.18% | 0.74% | 0.23% | 10.00% | 1.29% | 0.02% | 0.17% |
| 2017 | 0.25% | 0.09% | 9.87% | 0.22% | 0.92% | 0.25% | 9.52% | 1.43% | 0.02% | 0.19% |
| 2018 | 0.26% | 0.07% | 7.12% | 0.21% | 0.87% | 0.23% | 7.00% | 1.51% | 0.03% | 0.17% |
| 2019 | 0.30% | 0.08% | 14.02% | 0.18% | 0.80% | 0.25% | 7.81% | 0.86% | 0.02% | 0.19% |
| 2020 | 0.36% | 0.10% | 9.79% | 0.22% | 0.52% | 0.28% | 12.50% | 1.43% | 0.01% | 0.21% |
| 2021 | 0.33% | 0.08% | 11.30% | 0.17% | 0.53% | 0.22% | 9.68% | 1.08% | 0.02% | 0.19% |
| Average | 0.23% | 0.08% | 9.92% | 0.15% | 0.82% | 0.24% | 8.47% | 1.52% | 0.02% | 0.18% |

Supplementary Table S2. Distribution of Local GTWR Coefficients for Crime Rate (per 10,000 people)

| ***Dependent Variable*** | **Crime Rate**  **(Crime with Mental Illness per 10,000 people)** | | | | |
| --- | --- | --- | --- | --- | --- |
|  | **All** | **Violent** | **Normal** | **Larceny** | **Drug** |
| *Predictors* | *Median*  *[Q25, Q75]* | *Median*  *[Q25, Q75]* | *Median*  *[Q25, Q75]* | *Median*  *[Q25, Q75]* | *Median*  *[Q25, Q75]* |
| Year (Year2012 = 0) | 0 | 0 | 0 | 0 | 0 |
|  | [0.000, 0.000] | [0.000, 0.000] | [0.000, 0.000] | [0.000, 0.000] | [0.000, 0.000] |
| Clinical Psychologist (Clinic) | 1.675 | 0.688 | 1.182 | 0.698 | 0.017 |
|  | [0.736, 5.033] | [0.303, 1.791] | [1.177, 1.187] | [0.229, 1.574] | [−0.019, 0.043] |
| Counseling Psychologist (Clinic) | −1.135 | −0.318 | −0.526 | −0.358 | −0.017 |
|  | [−4.072, −0.106] | [−1.102, 0.015] | [−0.533, −0.521] | [−1.272, −0.145] | [−0.024, −0.005] |
| Clinical Social Worker (Clinic) | 0.535 | 1.104 | 0.043 | 0.794 | −0.029 |
|  | [−6.38e20, 7.31e20] | [−4.15e4, 5.83e4] | [0.041, 0.045] | [−6.38e12, 3.35e11] | [−0.029, −0.028] |
| Psychiatry (Clinic) | 0.114 | 0.086 | −0.283 | 0.015 | 0.037 |
|  | [−0.708, 1.968] | [−0.189, 0.892] | [−0.287, −0.276] | [−0.271, 0.391] | [0.022, 0.067] |
| Clinical Psychologist (Hospital) | −0.589 | −0.173 | −0.077 | −0.102 | 0.004 |
|  | [−0.945, −0.012] | [−0.436, −0.074] | [−0.089, −0.061] | [−0.256, 0.082] | [−0.005, 0.016] |
| Counseling Psychologist (Hospital) | 0.312 | 0.252 | −0.714 | −0.381 | −0.042 |
|  | [−3.186, 1.391] | [−0.843, 0.856] | [−0.760, −0.669] | [−1.097, 0.233] | [−0.099, −0.009] |
| Clinical Social Worker (Hospital) | 0.215 | 0.077 | −0.005 | 0.054 | 0.007 |
|  | [0.115, 0.422] | [0.035, 0.195] | [−0.009, 0.000] | [0.007, 0.176] | [0.005, 0.010] |
| Psychiatric Acute Bed | 0.042 | 0.018 | −0.014 | 0.013 | 0 |
|  | [−0.018, 0.108] | [−0.001, 0.052] | [−0.015, −0.014] | [−0.002, 0.033] | [0.000, 0.000] |
| Psychiatric Chronic Bed | −0.010 | −0.004 | 0 | −0.004 | 0 |
|  | [−0.032, 0.011] | [−0.009, 0.005] | [0.000, 0.001] | [−0.008, 0.003] | [0.000, 0.000] |
| Psychiatric Intensive Care Bed | 0.071 | 0.049 | 0.01 | −0.003 | −0.010 |
|  | [−0.731, 0.483] | [−0.284, 0.766] | [0.006, 0.013] | [−0.224, 0.100] | [−0.014, −0.007] |
| Psychiatry (Hospital) | −0.505 | −0.217 | 2.346 | −0.010 | 0.092 |
|  | [−2.477, 1.882] | [−0.694, 0.621] | [2.216, 2.472] | [−0.930, 0.923] | [0.082, 0.103] |
| Psychiatry Doctor | 0.076 | −0.015 | −0.007 | 0 | −0.012 |
|  | [−0.256, 0.277] | [−0.095, 0.095] | [−0.015, 0.000] | [−0.155, 0.061] | [−0.018, −0.009] |
| Low-Income Household | −0.001 | 0 | 0.001 | 0 | 0 |
|  | [−0.002, 0.000] | [−0.001, 0.000] | [0.001, 0.001] | [−0.001, 0.000] | [0.000, 0.000] |
| College Degree | 0 | 0 | 0 | 0 | 0 |
|  | [−0.001, 0.000] | [0.000, 0.000] | [0.000, 0.000] | [0.000, 0.000] | [0.000, 0.000] |
| Intercept | 0.009 | 0.033 | −0.015 | −0.001 | 0.001 |
|  | [−0.003, 0.052] | [0.024, 0.055] | [−0.016, −0.014] | [−0.020, 0.006] | [0.000, 0.001] |

Supplementary Table S3. Distribution of Local GTWR Coefficients for Mental Illness Case Proportion (per 100 cases)

| ***Dependent Variable*** | **Mental Illness Case Proportion**  **(Crime with Mental Illness per 100 cases)** | | | | |
| --- | --- | --- | --- | --- | --- |
|  | **All** | **Violent** | **Normal** | **Larceny** | **Drug** |
| *Predictors* | *Median*  *[Q25, Q75]* | *Median*  *[Q25, Q75]* | *Median*  *[Q25, Q75]* | *Median*  *[Q25, Q75]* | *Median*  *[Q25, Q75]* |
| Year (Year2012 = 0) | 0 | −0.004 | 0 | 0.003 | 0 |
|  | [0.000, 0.001] | [−0.009, −0.003] | [0.000, 0.000] | [0.002, 0.004] | [0.000, 0.000] |
| Clinical Psychologist (Clinic) | 7.271 | 9.062 | 2.393 | 4.779 | −0.210 |
|  | [5.605, 8.712] | [−0.775, 31.800] | [2.382, 2.400] | [2.038, 12.119] | [−1.179, 0.300] |
| Counseling Psychologist (Clinic) | −1.281 | −5.100 | −0.603 | −2.544 | −0.066 |
|  | [−1.418, −1.048] | [−10.940, −2.950] | [−0.622, −0.591] | [−5.516, −1.844] | [−0.298, 0.129] |
| Clinical Social Worker (Clinic) | 8.769 | 27.956 | 1.022 | 20.295 | −0.114 |
|  | [8.610, 9.167] | [23.770, 39.925] | [1.017, 1.033] | [−4.95e6, 7.82e6] | [−0.171, −0.092] |
| Psychiatry (Clinic) | −0.454 | 4.395 | −1.157 | −0.118 | 0.07 |
|  | [−0.714, 0.096] | [−0.476, 8.303] | [−1.178, −1.123] | [−1.319, 0.915] | [−0.031, 0.770] |
| Clinical Psychologist (Hospital) | −0.254 | −3.420 | −0.444 | −0.609 | −0.029 |
|  | [−0.472, −0.012] | [−6.860, −2.060] | [−0.468, −0.412] | [−1.564, 0.020] | [−0.122, 0.100] |
| Counseling Psychologist (Hospital) | −0.237 | 2.46 | −2.178 | −1.759 | −0.197 |
|  | [−4.411, 1.216] | [−28.461, 10.807] | [−2.257, −2.091] | [−6.285, 1.454] | [−0.857, 0.025] |
| Clinical Social Worker (Hospital) | 0.346 | 1.478 | −0.088 | 0.448 | 0.018 |
|  | [0.294, 0.385] | [1.148, 3.308] | [−0.102, −0.071] | [0.106, 1.067] | [−0.010, 0.055] |
| Psychiatric Acute Bed | 0.017 | 0.219 | −0.053 | 0.082 | 0 |
|  | [−0.015, 0.040] | [−0.054, 0.675] | [−0.055, −0.050] | [−0.012, 0.161] | [−0.003, 0.006] |
| Psychiatric Chronic Bed | 0.003 | 0.007 | 0 | −0.019 | 0 |
|  | [−0.005, 0.008] | [−0.072, 0.084] | [0.000, 0.000] | [−0.035, 0.016] | [−0.002, 0.000] |
| Psychiatric Intensive Care Bed | −0.020 | 1.434 | 0.278 | 0.132 | −0.015 |
|  | [−0.162, 0.078] | [−0.351, 5.553] | [0.250, 0.306] | [−0.393, 1.263] | [−0.049, 0.056] |
| Psychiatry (Hospital) | 0.681 | −2.220 | 8.662 | −0.176 | 0.527 |
|  | [0.193, 2.467] | [−10.339, 3.874] | [8.218, 9.067] | [−3.886, 7.252] | [0.390, 0.812] |
| Psychiatry Doctor | −0.268 | 0.384 | −0.047 | 0.017 | −0.019 |
|  | [−0.615, −0.022] | [−1.141, 1.533] | [−0.066, −0.030] | [−0.760, 0.311] | [−0.067, −0.013] |
| Low-Income Household | 0.001 | −0.001 | 0.001 | 0 | 0 |
|  | [−0.001, 0.002] | [−0.009, 0.002] | [0.001, 0.001] | [−0.004, 0.001] | [0.000, 0.000] |
| College Degree | 0 | 0 | 0.001 | −0.001 | 0 |
|  | [0.000, 0.000] | [−0.002, 0.002] | [0.001, 0.001] | [−0.001, 0.000] | [0.000, 0.000] |
| Intercept | −0.012 | 0.579 | 0.004 | −0.315 | 0.003 |
|  | [−0.026, 0.014] | [0.466, 0.978] | [0.001, 0.006] | [−0.394, −0.226] | [−0.002, 0.009] |

Supplementary Table S4. Distribution of Local GTWR Coefficients for Aggregated Mental Health Resources Predicting Crime Rate (per 10,000 People)

| ***Dependent Variable*** | **Crime Rate  (Crime with Mental Illness per 10,000 people)** | | | | |
| --- | --- | --- | --- | --- | --- |
|  | **All** | **Violent** | **Normal** | **Larceny** | **Drug** |
| *Predictors* | *Median*  *[Q25, Q75]* | *Median*  *[Q25, Q75]* | *Median*  *[Q25, Q75]* | *Median*  *[Q25, Q75]* | *Median*  *[Q25, Q75]* |
| Year (Year2012 = 0) | 0 | 0 | 0 | 0 | 0 |
|  | [−0.001, 0.000] | [0.000, 0.000] | [0.000, 0.000] | [0.000, 0.000] | [0.000, 0.000] |
| Psychologist & Social Worker | 0.054 | 0.023 | 0.031 | 0.021 | 0.007 |
|  | [−0.008, 0.209] | [−0.017, 0.087] | [0.025, 0.037] | [−0.018, 0.088] | [0.006, 0.010] |
| Psychiatry | −0.026 | −0.121 | 0.658 | −0.028 | 0.058 |
|  | [−1.004, 1.464] | [−0.293, 0.498] | [0.406, 1.002] | [−0.365, 0.492] | [0.048, 0.071] |
| Psychiatric Bed | −0.004 | 0 | 0.001 | −0.001 | 0 |
|  | [−0.019, 0.007] | [−0.004, 0.003] | [0.001, 0.001] | [−0.006, 0.002] | [0.000, 0.000] |
| Psychiatry Doctor | 0.09 | 0 | −0.061 | 0.014 | −0.014 |
|  | [−0.386, 0.253] | [−0.055, 0.056] | [−0.084, −0.044] | [−0.162, 0.043] | [−0.016, −0.012] |
| Low Income Family | −0.001 | 0 | 0 | 0 | 0 |
|  | [−0.002, 0.000] | [−0.001, 0.000] | [0.000, 0.001] | [−0.001, 0.000] | [0.000, 0.000] |
| College Degree | 0 | 0 | 0 | 0 | 0 |
|  | [−0.001, 0.001] | [0.000, 0.000] | [0.000, 0.000] | [0.000, 0.000] | [0.000, 0.000] |
| Intercept | 0.032 | 0.037 | −0.015 | 0.003 | 0.001 |
|  | [0.005, 0.089] | [0.028, 0.059] | [−0.018, −0.013] | [−0.017, 0.013] | [0.000, 0.001] |

Supplementary Table S5. Distribution of Local GTWR Coefficients for Aggregated Mental Health Resources Predicting Mental Illness Case Proportion (per 100 Cases)

| ***Dependent Variable*** | **Mental Illness Case Proportion**  **(Crime with Mental Illness per 100 cases)** | | | | |
| --- | --- | --- | --- | --- | --- |
|  | **All** | **Violent** | **Normal** | **Larceny** | **Drug** |
| *Predictors* | *Median*  *[Q25, Q75]* | *Median*  *[Q25, Q75]* | *Median*  *[Q25, Q75]* | *Median*  *[Q25, Q75]* | *Median*  *[Q25, Q75]* |
| Year (Year2012 = 0) | 0 | −0.005 | 0 | 0.003 | 0 |
|  | [0.000, 0.000] | [−0.009, −0.003] | [0.000, 0.000] | [0.002, 0.004] | [0.000, 0.000] |
| Psychologist & Social Worker | 0.205 | 0.292 | 0.025 | 0.144 | 0.029 |
|  | [0.158, 0.218] | [−0.172, 1.021] | [−0.004, 0.051] | [0.034, 0.433] | [0.027, 0.032] |
| Psychiatry | 0.251 | −1.321 | 2.693 | −0.256 | 0.275 |
|  | [−0.112, 1.868] | [−3.888, 1.964] | [2.016, 3.508] | [−1.782, 2.638] | [0.267, 0.280] |
| Psychiatric Bed | 0.001 | −0.007 | 0.001 | −0.010 | 0 |
|  | [−0.001, 0.002] | [−0.057, 0.057] | [0.001, 0.002] | [−0.022, 0.008] | [0.000, 0.000] |
| Psychiatry Doctor | −0.280 | 0.641 | −0.279 | −0.085 | −0.073 |
|  | [−0.369, −0.164] | [−0.519, 1.445] | [−0.334, −0.224] | [−0.715, 0.164] | [−0.073, −0.072] |
| Low Income Family | 0.001 | −0.002 | 0 | −0.001 | 0 |
|  | [0.000, 0.002] | [−0.011, 0.000] | [0.000, 0.000] | [−0.003, 0.001] | [0.000, 0.000] |
| College Degree | 0 | 0 | 0 | −0.001 | 0 |
|  | [0.000, 0.000] | [−0.002, 0.002] | [0.000, 0.000] | [−0.002, 0.000] | [0.000, 0.000] |
| Intercept | 0.003 | 0.713 | 0.005 | −0.314 | −0.003 |
|  | [−0.015, 0.024] | [0.498, 1.010] | [−0.001, 0.008] | [−0.394, −0.190] | [−0.004, −0.002] |


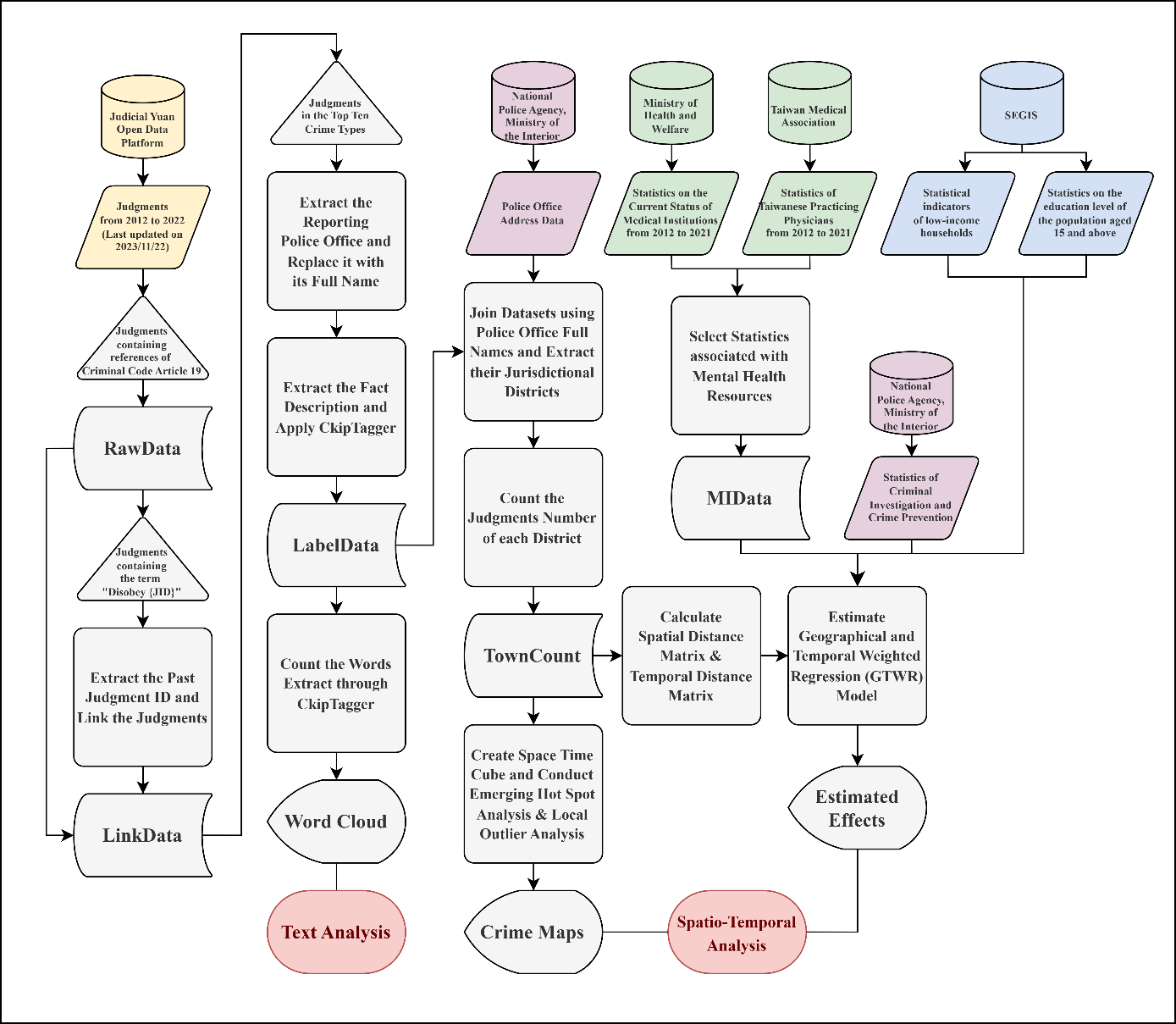


Supplementary Figure S1. Analytical Flowchart


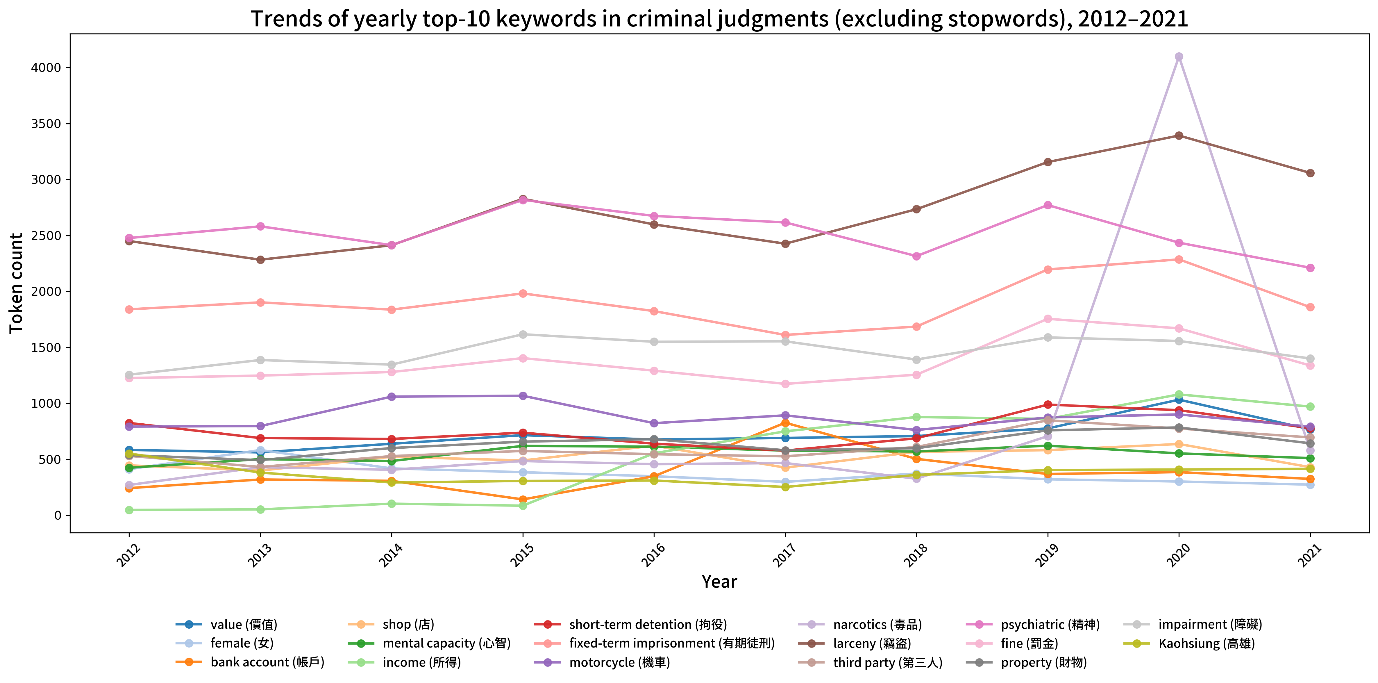


Supplementary Figure S2. Trends of Yearly Top-10 Keywords in Criminal Judgments (Excluding Stopwords), 2012-2021


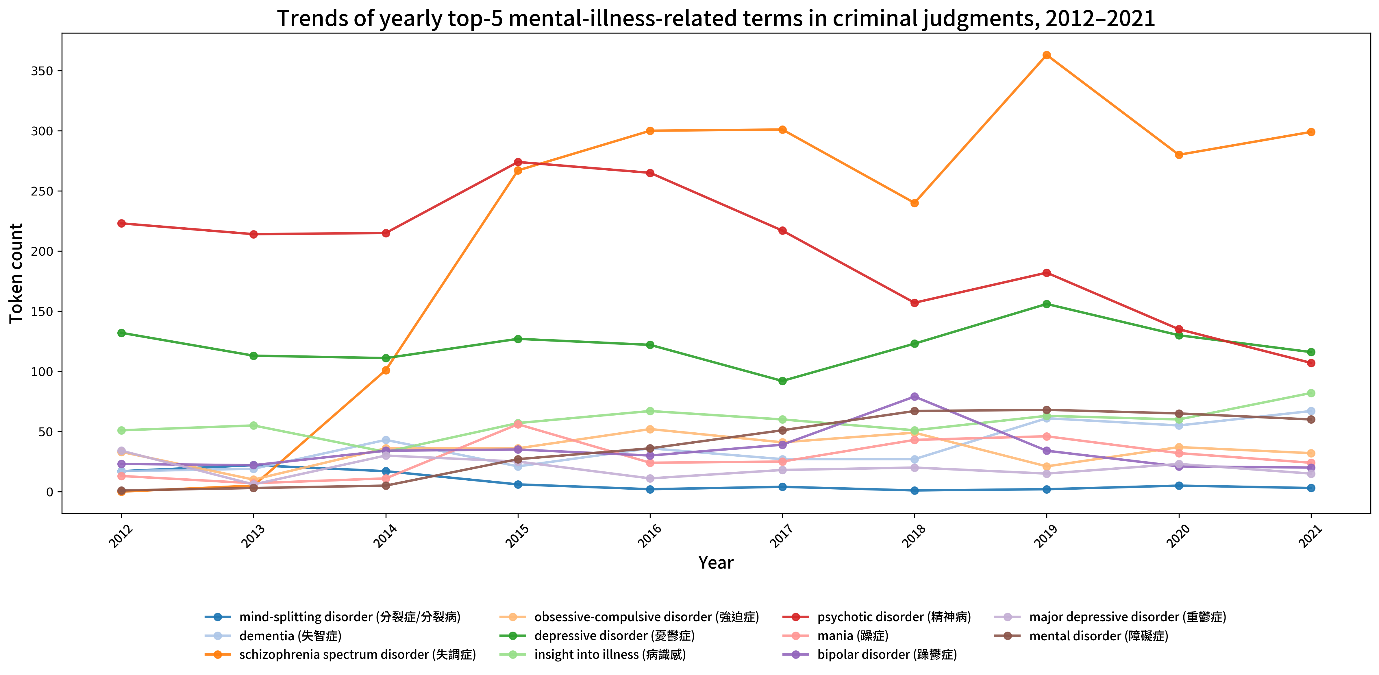


Supplementary Figure S3. Trends of Yearly Top-5 Mental-Illness-Related Terms in Criminal Judgments, 2012-2021


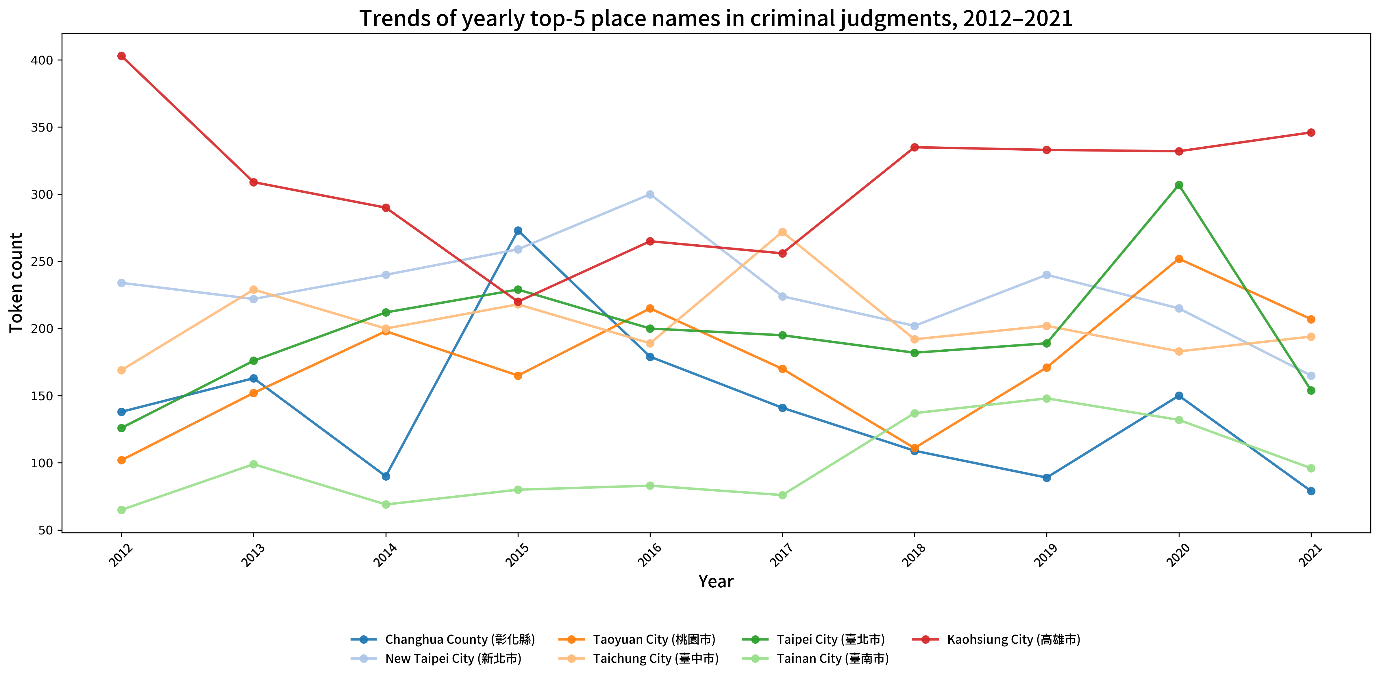


Supplementary Figure S4. Trend of Yearly Top-5 Place Names in Criminal Judgments, 2012-2021


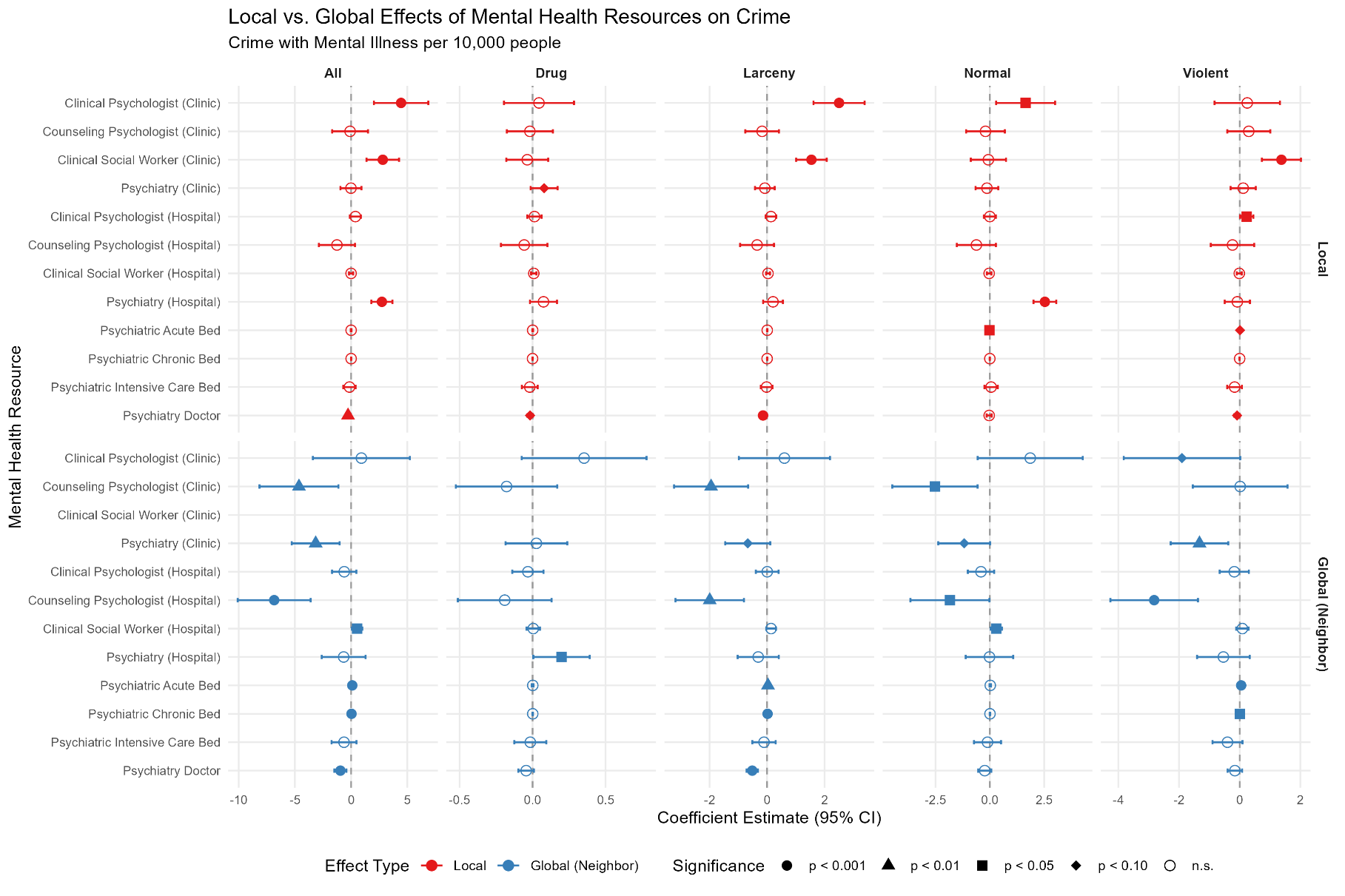


Supplementary Figure S5. Local and Global (Neighbor) Effects of Mental Health Resources on Crime with Mental Illness per 10,000 People Estimated Using OLS Spatial Lag Models


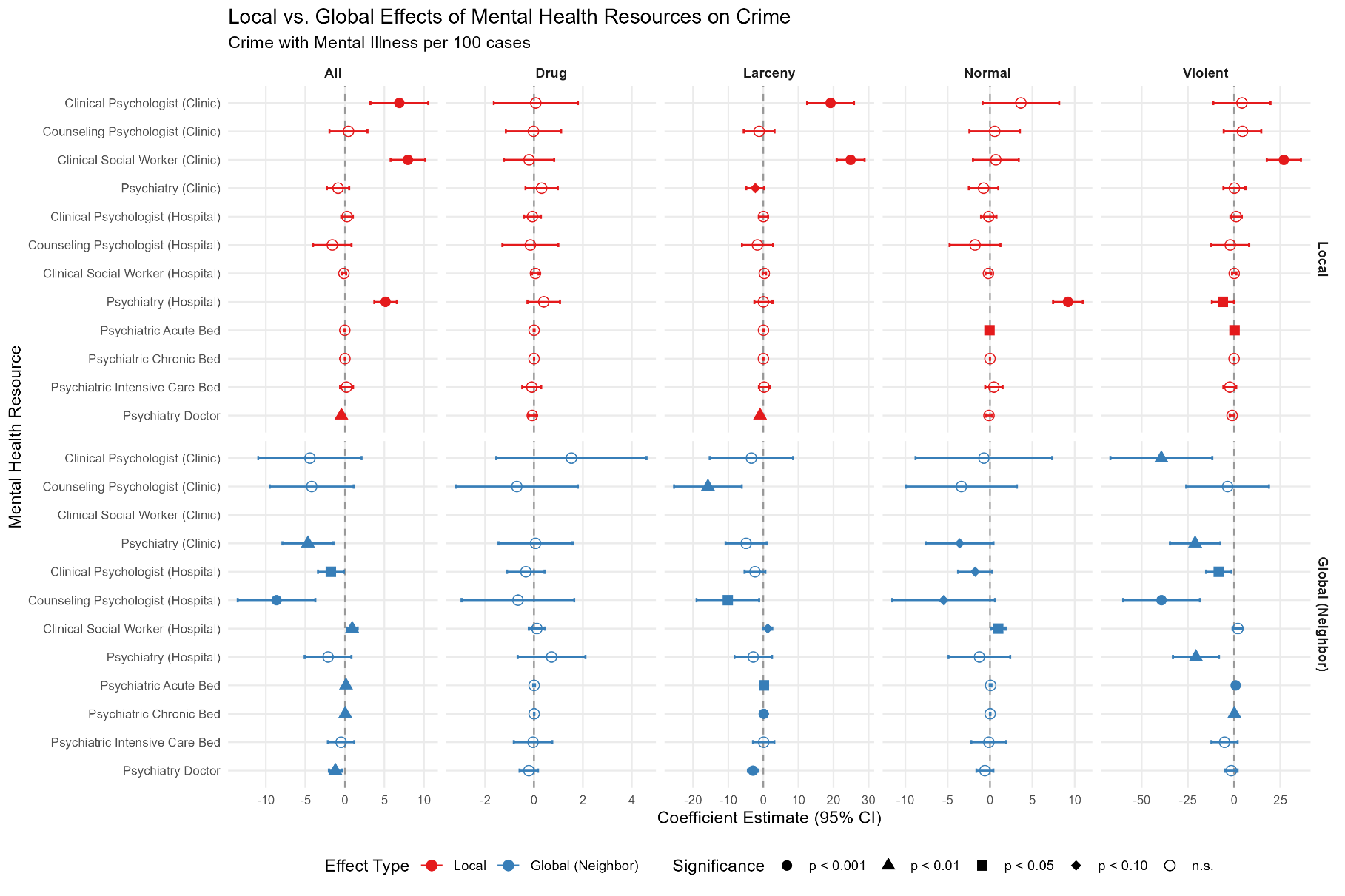


Supplementary Figure S6. Local and Global (Neighbor) Effects of Mental Health Resources on Crime with Mental Illness per 100 Cases Estimated Using OLS Spatial Lag Models


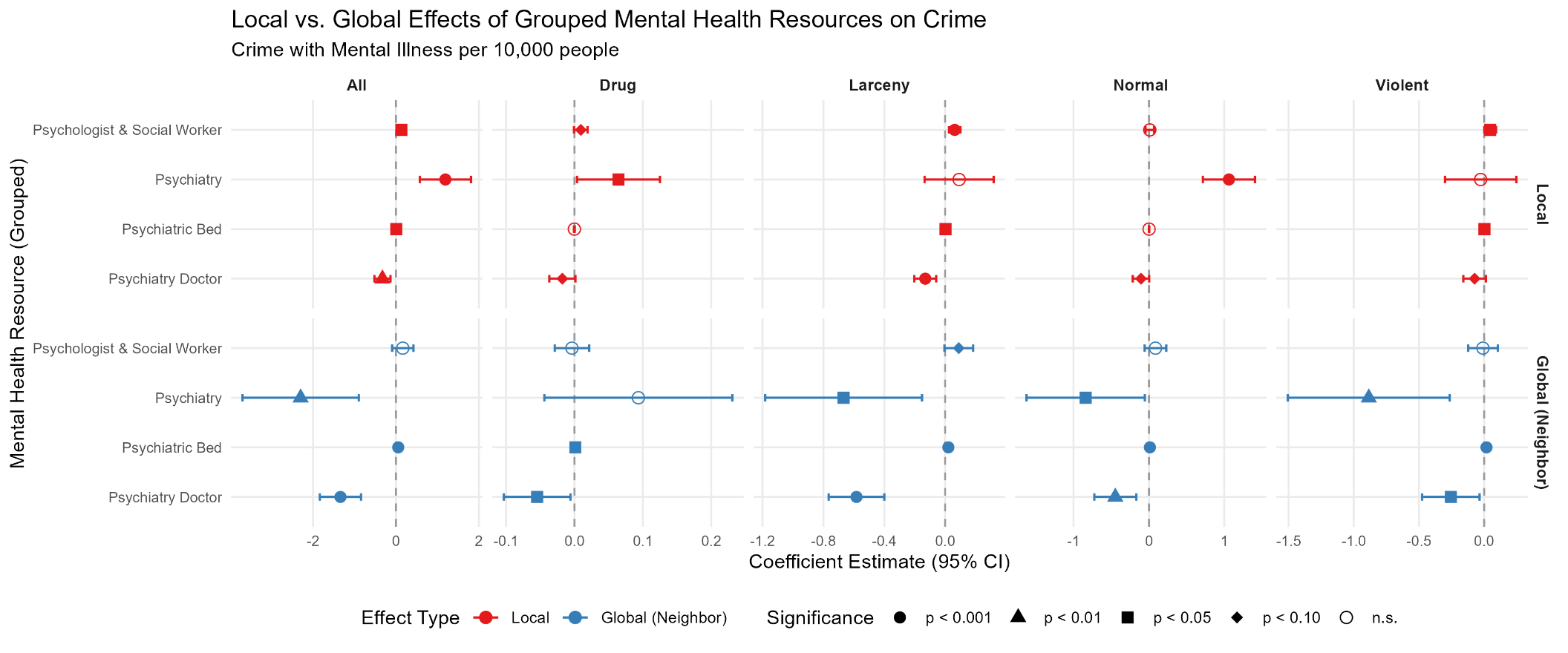


Supplementary Figure S7. Local and Global (Neighbor) Effects of Grouped Mental Health Resources on Crime with Mental Illness per 10,000 People Estimated Using OLS Spatial Lag Models


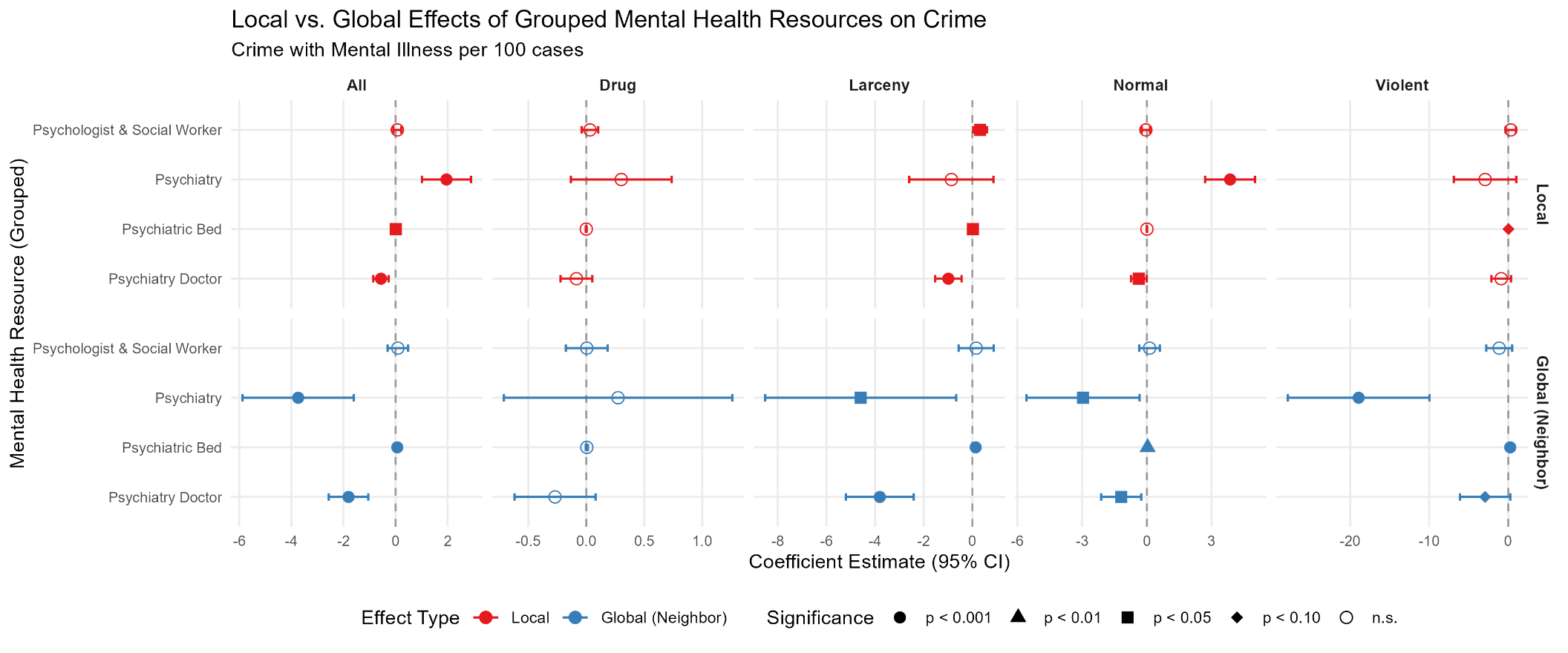


Supplementary Figure S8. Local and Global (Neighbor) Effects of Grouped Mental Health Resources on Crime with Mental Illness per 100 Cases Estimated Using OLS Spatial Lag Models
